# Downregulation of Linc00173 increases BCL2 mRNA stability via the miR-1275/PROCA1/ZFP36L2 axis and induces acquired cisplatin resistance of lung adenocarcinoma

**DOI:** 10.1186/s13046-022-02560-6

**Published:** 2023-01-10

**Authors:** Xingyu Tao, Yang Li, Songqing Fan, Liyang Wu, Jianyang Xin, Yun Su, Xiaoyang Xian, Yingying Huang, Rongquan Huang, Weiyi Fang, Zhen Liu

**Affiliations:** 1 Guangzhou Municipal and Guangdong Provincial Key Laboratory of Protein Modification and Degradation, State Key Laboratory of Respiratory Disease, Guangzhou Medical University, Guangzhou, 511436 China; 2 The Second Xiangya Hospital of Central South University, Changsha, 410008 China; 3 Cancer Research Institute, School of Basic Medical Sciences, Southern Medical University, Guangzhou, 510515 China; 4 Cancer Center, Integrated Hospital of Traditional Chinese Medicine, Southern Medical University, Guangzhou, 510315 China; 5 Department of Pathology, Guangzhou Municipal and Guangdong Provincial Key Laboratory of Protein Modification and Degradation, State Key Laboratory of Respiratory Disease, School of Basic Medical Sciences, Guangzhou Medical University, Guangzhou, 511436 China

**Keywords:** Lung adenocarcinoma, LINC00173, PROCA1, ZFP36L2, Apoptosis, BCL2 stability

## Abstract

**Background:**

LINC00173 had been reported as a cisplatin (*cis*-diamminedichloroplatinum, DDP) chemotherapy-resistant inducer in small-cell lung cancer (SCLC) and lung squamous cell carcinoma (LUSC). This study aimed to display reverse data for LINC00173 as a DDP chemosensitivity-inducing factor in lung adenocarcinoma (LUAD).

**Methods:**

LINC00173 was screened from the Gene Expression Omnibus database (GSE43493). The expression level of LINC00173 in LUAD tissues and cell lines was detected using in situ hybridization and quantitative reverse transcription–polymerase chain reaction. Colony formation, cell viability, half-maximal inhibitory concentration, flow cytometry, and xenograft mouse model were used to evaluate the role of LINC00173 in the chemosensitivity of LUAD to DDP. The mechanism of LINC00173 in DDP resistance by mediating miR-1275/PROCA1/ZFP36L2 axis to impair BCL2 mRNA stability was applied, and co-immunoprecipitation, chromatin immunoprecipitation, RNA antisense purification, RNA immunoprecipitation, and luciferase reporter assays were performed.

**Results:**

LINC00173 downregulation in patients with DDP-resistant LUAD was correlated with poor prognosis. Further, LINC00173 expression was significantly reduced in DDP-resistant LUAD cells and DDP-treated human LUAD tissues. Suppressed LINC00173 expression in LUAD cells enhanced DDP chemoresistance in vivo and in vitro, while restored LINC00173 expression in DDP-resistant LUAD cells markedly regained chemosensitivity to DDP. Mechanistically, DDP-resistant LUAD cells activated PI3K/AKT signal and further elevated the c-Myc expression. The c-Myc, as an oncogenic transcriptional factor, bound to the promoter of LINC00173 and suppressed its expression. The reduced LINC00173 expression attenuated the adsorption of oncogenic miR-1275, downregulating the expression of miR-1275 target gene PROCA1. PROCA1 played a potential tumor-suppressive role inducing cell apoptosis and DDP chemosensitivity via recruiting ZFP36L2 to bind to the 3′ untranslated region of BCL2, reducing the stability of BCL2 mRNA and thus activating the apoptotic signal.

**Conclusions:**

This study demonstrated a novel and critical role of LINC00173. It was transcriptionally repressed by DDP-activated PI3K/AKT/c-Myc signal in LUAD, promoting DDP-acquired chemotherapeutic resistance by regulating miR-1275 to suppress PROCA1/ZFP36L2-induced BCL2 degradation, which led to apoptotic signal reduction. These data were not consistent with the previously described role of LINC00173 in SCLC or LUSC, which suggested that LINC00173 could play fine-tuned DDP resistance roles in different pathological subtypes of lung cancer. This study demonstrated that the diminished expression of LINC00173 might serve as an indicator of DDP-acquired resistance in LUAD.

**Supplementary Information:**

The online version contains supplementary material available at 10.1186/s13046-022-02560-6.

## Background

Lung cancer is the leading cause of cancer mortality worldwide because of its the poor survival and high relapse rates after surgery [1]. It is commonly divided into non-small-cell lung cancer (NSCLC) and small-cell lung cancer (SCLC) based on its clinical features and pathological classification [2]. NSCLC is the predominant type of lung cancer, accounting for 85% of all lung cancer cases. Histologically, it consists mainly of lung squamous cell carcinoma (LUSC) and lung adenocarcinoma (LUAD). The incidence of LUAD has increased in recent years, gradually replacing LUSC as the most common histological subtype [2]. Cisplatin (*cis*-diamminedichloroplatinum, DDP)-based chemotherapy is used as one of the primary treatments for intermediate and advanced LUAD [3]. Unfortunately, the acquired chemoresistance to DDP remains a major cause for poor therapeutic outcomes of LUAD [4–7]. Therefore, the mechanism of acquired resistance to DDP in LUAD needs to be urgently clarified.

In previous studies, some long noncoding RNAs (lncRNAs) with dysregulated expression levels have been reported to be involved in the acquired drug resistance of neoplasms [8–16]. LINC00173 was first introduced in 2017, located on human chromosome 12q24.22 and with a full length of 1597 bp [17]. Some studies showed that LINC00173 played a dual role in tumors, suggesting its complex role in the pathogenesis of malignancies [18–20]. Recently, LINC00173 has been to be upregulated in SCLC and LUSC, promoting resistance to chemotherapeutic drugs [21, 22]. However, its role and mechanism in DDP resistance in LUAD have not been elucidated.

In this study, we performed a detailed investigation of LINC00173 in DDP-based drug resistance in LUAD. The findings clarified the downregulation of LINC00173 and its chemosensitive role in DDP-resistant LUAD through a newly discovered pathway, indicating a potential clinical therapeutic target in LUAD, which was distinctly different from the role of LINC00173 in SCLC and LUSC.

## Materials and Methods

### Cell lines and cell culture

The five human LUAD cell lines (H1299, H1650, H1975, A549, SPCA1) used in this study were obtained from the cell bank of the Chinese Academy of Sciences (Shanghai, China). The PC9 cell line was kindly provided by Sun Yat-Sen University Cancer Center (Guangzhou, China). Immortalized non-malignant human bronchial epithelial cells 16HBE were obtained from the Cancer Research Institute of Southern Medical University (Guangzhou, China). In this study, all the cells were cultured in RPMI-1640 medium supplemented with 10% fetal bovine serum (FBS, PAN, USA), 100 units/mL penicillin, and 100 mg/mL streptomycin and kept in a humidified atmosphere containing 5% CO_2_ at 37 °C. To establish DDP-resistant A549 and PC9 cell lines, cells were treated with increasing concentrations of DDP (Sigma) from the initial concentration of 0.25 μM to the final concentration of 6 μM. DDP was added to the RPMI-1640 culture media for A549/DDP cells and PC9/DDP cells to maintain their DDP-resistant phenotype (with a final concentration of 6 μM).

### miRNA (mimics and inhibitor), siRNAs, plasmid, and lentivirus transfection

The LINC00173 Smart Silencer (RiboBio, Guangzhou, China) was a mixture of three small-interfering RNAs (siRNAs) with three antisense oligonucleotides that target different sites of the LINC00173 transcript. Compared with the LINC00173 Smart Silencer, the negative control Smart Silencer did not contain domain sequences homologous to humans. The other siRNAs (including three si-ZFP36L2 fragments) and microRNAs (miRNAs, including miR-1275 mimics and miR-1275 inhibitor) were also designed and synthesized by RiboBio. Transient transfections of all miRNAs and siRNAs were performed using the riboFECT™ CP reagent following the manufacturer’s instructions. PROCA1 plasmid was established by Vigene Bioscience, Inc. (Shandong, China). The human full-length LINC00173 was polymerase chain reaction (PCR)-amplified from cDNA and cloned into the CMV-MCS-IRES-EGFP-SV40-Neomycin overexpression vector (GV146, Genechem, China). All reporter plasmids used in the luciferase reporter assay were obtained from IGE Biotechnology Ltd (Guangzhou, China). The transient transfection of plasmids was conducted using Lipofectamine 3000 (Invitrogen, USA). The short hairpin RNA (shRNA) for human LINC00173 was cloned into a hU6-MCS-Ubiquitin-EGFP-IRES-puromycin lentiviral vector (GV248, Genechem, China). The sequences of Smart-Silencer, siRNAs or shRNA, miR-1275 mimics, miR-1275 inhibitor and their corresponding negative control are listed in Supplementary Table 1. All lentiviral particles were provided by Shanghai Genechem Co., Ltd. For lentiviral infection, 2 × 10^4^ cells were seeded into 24-well plates and infected with proper lentivirus or control lentivirus (as a negative control) at a multiplicity of infection of 20. The stably transduced cells were screened using puromycin (3 μg/mL; Meilunbio, Dalian, China), and the infection efficiency was assessed using a fluorescence microscope (Nikon, Japan).

### Patients and tissue specimens

Paraffin-embedded tissue samples from 129 patients with LUAD treated with platinum-based multidrug chemotherapy were used as tissue microarray (TMA), and were obtained from the Second Xiangya Hospital of Central South University (Hunan, China). The clinicopathological features of 129 patients with LUAD are summarized in Supplementary Table 2. Data regarding tumor stage was determined according to the pathological tumor-node-metastasis staging system (AJCC/UICC 2015). The histological types were determined according to the World Health Organization classification for LUAD. According to previous studies [21, 23, 24], the aforementioned patients with LUAD were defined as either platinum -resistant or platinum -sensitive.

### In situ hybridization

The expression level of LINC00173 in 129 paraffin-embedded LUAD specimens was detected by in situ hybridization (ISH). The TMA sections were dewaxed in xylene, rehydrated through an ethanol gradient, and then treated with 3% H_2_O_2_ for 10 min. Subsequently, the sections were treated with pepsin dilution in 3% fresh citrate buffer at 37 °C for 30 min and then washed with phosphate-buffered saline (PBS). LINC00173 digoxygenin-labeled probes were designed and synthesized by BersinBio (Bersin Biotechnology Co. Ltd, Guangzhou, China) (probe sequence: 5′-Dig-CGTGATCTGAGTACATGTAGGATAAATGACCCCAGGCAAGGC-3′). Further, hybridization was performed using LINC00173 probes at 75 °C for 5 min, quickly transferred to 42 °C, and incubated overnight. Then, the sections were incubated with anti-digoxygenin (HRP) Fab fragments at 37 °C for 1 h. All slides were scanned using the iViewer scanning system (UNIC, Beijing, China) and quantified using a staining index (ranging from 0 to 9), which was evaluated by multiplying the percentage of positively stained cells (0: 0%–25%; 1: 26%–50%; 2: 51%–75%; 3: 76%–100%) and staining intensity (0: negative; 1: weak; 2: moderate; and 3: strong) [25, 26]. The staining index of each sample was scored by two independent pathologists and averaged.

### Quantitative real-time reverse transcription–PCR

The cellular RNA was extracted from LUAD cells using the TRIzol reagent (Thermo Scientific, USA) and then reverse transcribed to cDNA using a Quantscript reverse transcription (RT) Kit (Takara, Japan). The quantitative RT-PCR (qRT-PCR) analysis was performed using an AriaMx Real-Time PCR (Agilent, USA) system following standard procedures. Glyceraldehyde-3-phosphate dehydrogenase (GAPDH) was used as a loading control, and the primer sequence was as follows: forward: 5′-CGGAGTCAACGGATTTGGTCGTAT-3′, reverse: 5′-AGCCTTCTCCATGGTGGTGAAGAC-3′. The other primers used for the qRT-PCR analyses are listed in Supplementary Table 3.

### Western blot analysis

The Western blot analysis was performed as described in a previous study [27]. The blotting bands were then incubated with primary antibodies overnight at 4 °C. The antibodies against the following proteins were used: Caspase-3 (Cell Signaling Technology #14220, 1:1000), PARP (Cell Signaling Technology #9542, 1:1000), BCL2 (Cell Signaling Technology #15071, 1:1000), PROCA1 (Biorbyt #orb1703, 1:1000), ZFP36L2 (Santa Cruz #sc-365908, 1:1000), c-Myc (Cell Signaling Technology #9402, 1:1000), phosphor-Akt (Cell Signaling Technology #4060, 1:1000), Akt (Cell Signaling Technology #4691, 1:1000), phosphor-PI3K (Cell Signaling Technology #17366, 1:1000), and PI3K (Cell Signaling Technology #4292, 1:1000). The internal reference antibodies were as follows: GAPDH (Cell Signaling Technology #5174, 1:1000) and β-tubulin (Proteintech #10068-1-AP, 1:1000). The secondary antibodies were as follows: HRP-conjugated Affinipure goat anti-rabbit immunoglobulin G (IgG) (H + L) (Proteintech #SA00001-2, 1:5000), and HRP-conjugated Affinipure goat anti-mouse IgG (H + L) (Proteintech #SA00001-1, 1:5000) were purchased from Proteintech (IL, USA). The images were captured by chemiluminescence imaging (Bio-Rad, CA, USA) and quantitated using the Quantity One system (Bio-Rad).

### Colony formation assay

The cells at a density of 200 cells/well were seeded in six-well plates. For the DDP-treated group, a medium containing 2 μM or 6 μM DDP was added, respectively, to the parental cells (A549 and PC9) or DDP-resistant cells (A549-DDP and PC9-DDP) after cell adhesion. As the transduction of inhibitors or mimics of miR-1275, plasmids of PROCA1 or LINC00173 was transient, we transducted them every three days in the 6-well plate to maintain their expression. The generated colonies were cultured for 10–14 days and then fixed with methanol for 30 min. Subsequently, the colonies were stained with a crystal violet solution. The colonies composed of more than 50 cells in a well were counted using a microscope.

### Cell viability and half-maximal inhibitory concentration assays

For the cell viability assays, 2 × 10^4^ LUAD cells were seeded into 96-well plates. DDP was added after cell attachment, and the cell viability was detected using a cell counting kit-8 (CCK-8) reagent after 72 h. The absorbance at 450 nm measured in cell-free wells was used as blank.

For half-maximal inhibitory concentration (IC_50_) assays, 1–2 × 10^4^ LUAD cells were inoculated into a 96-well plate. A medium containing an increasing concentration of DDP (0 μM, 1 μM, 2 μM, 4 μM, 8 μM, 16 μM, 32 μM, and 64 μM) was added to the wells after cell attachment. Then, the plate was incubated for 48 h in a humidified atmosphere containing 5% CO_2_ at 37 °C. The analysis was performed following the instructions of the CCK-8 manufacturer, and the IC_50_ value of DDP was calculated using the GraphPad Prism v5.0 software (GraphPad Software Inc., CA, USA).

### Apoptosis

Annexin-V/propidium iodide (PI) was applied to quantify the number of apoptotic cells. The cells transfected with Smart Silencer for LINC00173 depletion or negative control were exposed to 3 μM DDP for 24 h. Both floated and adherent cells were collected, resuspended in binding buffer, and stained with FITC–Annexin-V and PI (BestBio, Shanghai, China). The stained cells were quantified using a flow cytometer (Biosciences, NJ, USA) following the manufacturer’s instructions.

### Luciferase reporter assays

The online software DIANA (http://diana.imis.athena-innovation.gr/) predicted that miR-1275 bound to LINC00173, as shown in Fig. 3d. The RNA sequence of LINC00173 containing the putative wild-type (WT) binding sites or mutant-type (MUT) binding sites for miR-1275 was inserted into the psiCHECK-2 vector to construct the luciferase reporter vector psiCHECK-2-LINC00173-WT or psiCHECK-2-LINC00173-MUT (named as LINC00173-WT or LINC00173-MUT, respectively). PROCA1 was predicted to be directly regulated by miR-1275 using the prediction software (TargetScan), as shown in Fig. 3i. The predicted 3′ untranslated region (3′-UTR) fragment of PROCA1 recognized by miR-1275 was inserted into the psiCHECK-2 vector (named as “PROCA1-WT”). The GeneTailor system (Invitrogen) was used to perform the site-directed mutagenesis of the miR-1275-binding site at the PROCA1 3′-UTR (named as “PROCA1-MUT”). Subsequently, the cells were co-transfected with WT or MUT psi-CHECK2 vector and miR-1275 or miR-inhibitor. The 2000 bp upstream of LINC00173 transcriptional start site was analyzed by PROMO (http://alggen.lsi.upc.es/cgi-bin/promo_v3/promo/promoinit.cgi?dirDB=T-F_8.3) to identify the possible binding transcriptional factors. A c-Myc-binding site was found in the promoter region of LINC00173 (Fig. 7a). The LINC00173 promoter sequences containing WT or MUT of c-Myc-binding sites were synthesized and constructed into the psiCHECK-2 vector (named as LINC00173-WT or LINC00173-MUT). Then, LINC00173-WT or LINC00173-MUT was, respectively, co-transfected into A549 cells together with the c-Myc or control plasmid. The luciferase activity was measured 48 h after transfection using the dual-luciferase reporter assay (Promega Corporation, WI, USA) kit following the manufacturer’s instructions. The detected relative luciferase activity was normalized to the Renilla luciferase activity.

### RNA antisense purification and RNA immunoprecipitation assays

The RNA antisense purification (RAP) assay was performed using an RAP Kit (Axl-bio, Guangzhou, China). We cross-linked 1 × 10^8^ cells to fix endogenous RNA complexes and then purified these complexes through hybrid capture with biotinylated antisense oligos. According to the number of probes (probe sequence: AGCGTGTGCAGGACTTAGCTTTG CTCTTGCACTGAGA; ATAGAAGGTCCCCCACGGGGACTTGGGAAGGCAC AGAGAACGCTCCCT; ATAAACAAGCTGTGACAGGTGATCATTCATCATAT TCCGCTGAACGTTCCA), streptavidin beads were prepared for RAP and negative control (NC) groups. A high-sensitivity DNA kit (Vazyme Biotech Co., Ltd, Q311) was used to examine the DNA fragment sizes. The extracted RNA was then verified by qRT-PCR.

The RNA immunoprecipitation (RIP) assay was performed using a Magna RIP RNA Binding Protein Immunoprecipitation Kit (Millipore, MA, USA; #MAGNARIP01). Then, 1 × 10^7^ cells were cultured in 75-cm^2^ cell culture flasks and harvested using a RIP lysis buffer. The ZFP36L2 antibody (Santa Cruz #sc-365908) or normal mouse IgG (negative control) were pre-incubated with magnetic beads to form a magnetic bead–antibody complex. Subsequently, the cell lysates were incubated with the bead–antibody complex overnight at 4 °C. The extracted RNA was then verified by qRT-PCR. All steps of RAP and RIP assays were performed following the kit manufacturer’s instructions.

### ChIP assay

A ChIP assay kit (Thermo, MA, USA, #26156) was used to determine whether c-Myc bound to the promoter of LINC00173 in A549 and A549-DDP cells, following the manufacturer’s instructions. The micrococcal nuclease was used to cut the cross-linked DNA to a length of 200–500 base pairs, which was then subjected to an immunoselection process requiring the use of an anti-c-Myc antibody (Cell Signaling Technology #13987). Ultimately, the agarose gel electrophoresis results demonstrated whether the DNA fragment of the putative c-Myc binding site was present in the LINC00173 promoter.

### Co-IP assay

A Co-IP kit (Thermo, MA, USA, #26149) was used to detect whether PROCA1 bound to ZFP36L2, following the manufacturer’s instructions. The proteins were extracted from the parental cells (A549 and PC9) or DDP-resistant cells (A549-DDP and PC9-DDP), and their concentration was quantified. A total of 5 mg of protein was incubated with 10 μg of specific ZFP36L2 antibody (Santa Cruz #sc-365908) or IgG control overnight at 4 °C. After elution, the proteins binding with ZFP36L2 were subjected to Western blot analysis. IgG was used as a negative control. One percent of the sample was used as input.

### Immunofluorescence

For the Immunofluorescence assay, 100–200 cells were seeded on a glass-bottom dish. After 48 h of culture, cells were washed with PBS and fixed in 4% paraformaldehyde for 15 min. The cells were permeabilized using 0.1% Triton X-100 in PBS before antibody incubation. Images were captured using a Zeiss confocal fluorescence microscope (LSM 800, Oberkochen, Germany) and ZenPro software 2011 (AZoM.com Limited, UK). Antibodies used in this assay were listed in the methods for Western Blot analysis.

### Animal experiments

Twenty female BALB/c nude mice (3–4 weeks old) were randomly divided into two groups. A549 NC control cells or A549 shLINC00173-1 cells (6 × 10^6^ cells) in 50 μL of PBS mixed with 50 μL of Matrigel matrix (Corning, USA, #354234) were injected subcutaneously into the flanks. Subsequently, the mice were monitored for xenograft development every other day. Next, 10 mice in the A549 NC group or A549 shLINC00173-1 group were randomly divided into two subgroups when the tumors reached a diameter of 5 mm in size and treated, respectively, with an intraperitoneal injection of DDP (3 mg/kg) or an equal volume (100 μL) of normal saline (N.S) every 2 days for 2 weeks. The mice were sacrificed by cervical dislocation, and the xenograft tumors resected from mice were weighed and examined with routine tissue processing. Animal experiments were performed in three independent replicates. All animal experiments were conducted according to standards regarding the use of laboratory animals. The protocols for animal experiments complied with the requirements of the Institutional Animal Ethical Committee, Experimental Animal Center of Guangzhou Medical University, China.

### Statistical analysis

All data were analyzed using the SPSS16.0 software (IBM Corp., NY, USA) and GraphPad Prism v7.0 software. Data were presented as mean ± SD. Statistical significance was determined using the Student’s two-tailed t-test between two groups and one-way analysis of variance (ANOVA) for multiple groups. ISH analysis results were analyzed by the chi-square (χ^2^) test. Survival analysis was performed using the Kaplan-Meier method. *P* value < 0.05 indicated statistical significance (*P < 0.05, **P < 0.01, ***P < 0.001, ****P < 0.0001).

## Results

### Downregulated LINC00173 expression in patients with DDP-resistant LUAD correlated with poor prognosis

**Fig. 1:**
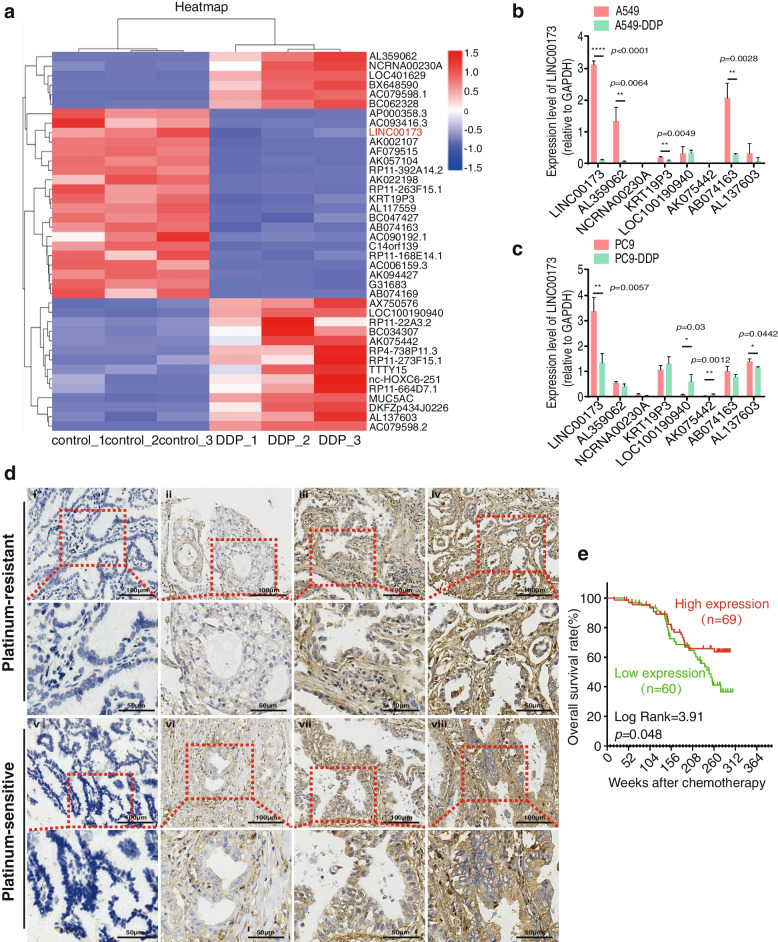
Decreased LINC00173 is unfavorable for DDP-resistant LUAD patients. a. Hierarchical clustering analysis of GEO dataset (GSE43493) displaying differentially expressed lncRNAs (including LINC00173) between A549 and A549-DDP cells. b and c. QRT-PCR analyses of eight lncRNAs (including LINC00173) in parental cells (A549 and PC9) and DDP-resistant cells (A549-DDP and PC9-DDP). Student’s two-tailed t-test, *P < 0.05; **P < 0.01; ****P < 0.0001. d. In situ hybridization showing the expression level of LINC00173 in platinum-resistant and platinum-sensitive patients. Figures i, ii, v and vi represent negative or low expression, iii, iv, vii and viii represent high expression (Scale bars, 100 μm). e. Kaplan–Meier survival curve showing overall survival of 129 LUAD patients based on LINC00173 expression. Log-rank test was utilized for *P* value calculation. Error bars, mean ± SD

We used bioinformatics analyses of the RNA-seq data (GSE43493) of the Gene Expression Omnibus database to screen the differentially expressed lncRNAs between LUAD DDP-resistant A549-DDP cells and their parental A549 cells to investigate the specific mechanism of DDP-based resistance in LUAD. Eight lncRNAs were found to exhibit the most significant difference (Fig. 1a). DDP-resistant A549-DDP and PC9-DDP cell lines were established by gradually dosing A549 and PC9 cells with DDP in vitro until their IC_50_ value significantly increased. The resistance index of two resistant cell lines was 2.18 (A549-DDP/A549) and 2.06 (PC9-DDP/PC9) (Supplementary Fig. 1a and 1b). Subsequently, qRT-PCR detection identified that LINC00173 was the most significantly downregulated lncRNA in both A549-DDP- and PC9-DDP-resistant cells compared with their parental A549 and PC9 cells (Fig. 1b and 1c). We further examined LINC00173 expression using ISH in a restricted cohort of locally advanced LUAD treated with DDP-based multidrug chemotherapy to evaluate the association between LINC00173 dysregulation and the response to DDP-based chemotherapy in patients with LUAD. As shown in Fig. 1d, LINC00173 was mainly expressed in the cytoplasm. Of all the 129 patients, 86 were defined as platinum resistant, whereas the other 43 patients were evaluated as platinum sensitive. Low expression of LINC00173 was observed in 46/86 platinum-resistant cases (53.5%) and in only 14/43 platinum-sensitive cases (32.6%). A significant association was found between LINC00173 low expression and chemotherapeutic resistance in patients with LUAD (*P* = 0.025, Supplementary Table 4). Notably, the Kaplan-Meier survival analysis showed that patients with LUAD with low expression of LINC00173 had poorer overall survival (*P* = 0.048, Fig. 1e). The LINC00173 expression level was also detected in various LUAD cell lines (H1299, H1650, H1975, A549, PC9, and SPCA1) and immortalized human bronchial epithelial cells 16HBE. Interestingly, contrary to its expression level in 16HBE cells, the endogenous expression of LINC00173 showed inconsistency in diverse LUAD cell lines (Supplementary Fig. 1c). Taken together, our data suggested that LINC00173 was downregulated in DDP-resistant LUAD and contributed to the chemotherapeutic resistance and poor prognosis of patients with LUAD.

### Targeting LINC00173 desensitized LUAD cells to DDP treatment in vitro and in vivo

**Fig. 2:**
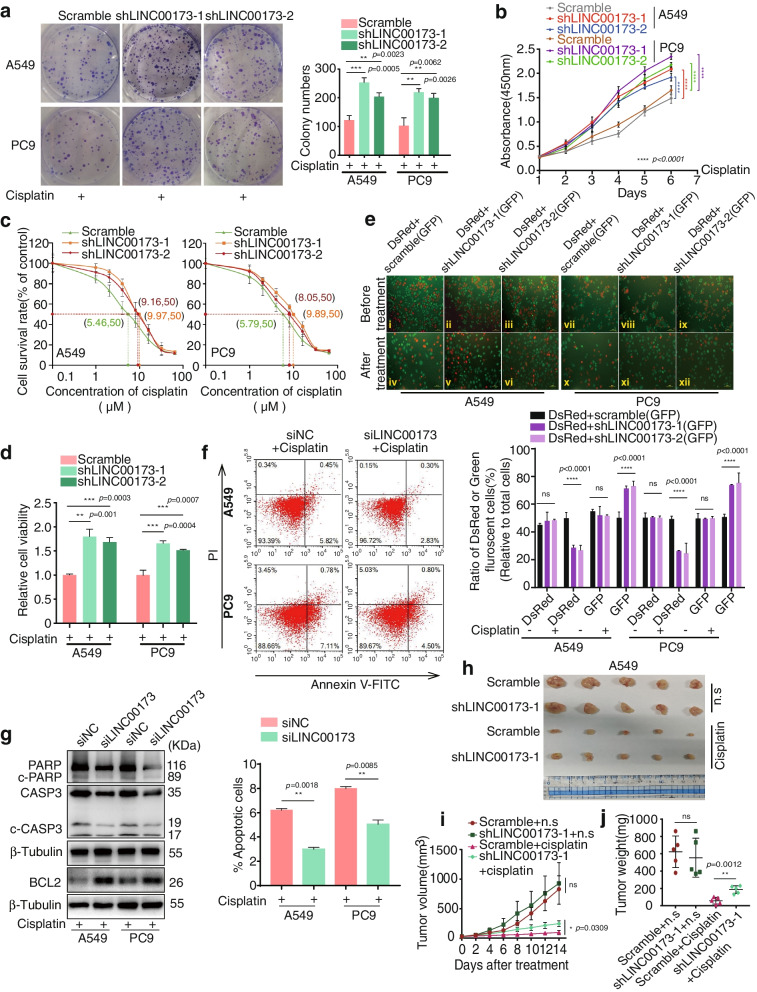
LINC00173 inhibition attenuates the chemotherapeutic sensitivity of LUAD cells to DDP in vitro and in vivo. a. Colony formation assays were conducted to determine the colony numbers of LINC00173 downregulated A549 and PC9 cells with cisplatin treatment. Student’s two-tailed t-test, **P < 0.01; ***P < 0.001. b, c and d. CCK8 assays were performed to detect the cell growth (b), IC_50_ values (c) and cell viability (d) of LINC00173 silenced A549 and PC9 cells with cisplatin treatment. Student’s two-tailed t-test, **P < 0.01; ***P < 0.001; ****P < 0.0001. e. Fluorescence images of LINC00173 downregulated cells (shLINC00173-1/2) and their control parental cells (A549/PC9) before or after cisplatin treatment (10 μM). shLINC00173-1/2 or control shRNAs were labeled with GFP; Parental A549/PC9 cells were labeled with DsRed. Figures i, ii, iii and vii, viii, ix exhibit the mixture of an equal number of DsRed and GFP cells. Figures iv, v, vi and x, xi, xii represent the corresponding cisplatin treated cells (scale bar, 100 μm). One-way analysis of variance, ****P < 0.0001; ns, no significance. f. The effect of LINC00173 downregulation on cisplatin-induced apoptosis of A549 and PC9 cells detected by flow cytometry. Student’s two-tailed t-test, **P < 0.01. g. Expression levels of PARP, cleaved-PARP (c-PARP), Caspase3, cleaved-Caspase3, and BCL2 were determined by western blot in LINC00173-interfered A549 and PC9 cells which cultured with medium containing cisplatin. β-Tubulin was used as a loading control. h, i and j. Effect of LINC00173 on LUAD chemosensitivity to cisplatin in vivo. Growth curves (i) and weight of tumor xenografts (j) generated from A549 cells expressing either shRNAs targeting LINC00173 (shLINC00173-1) or nontargeting shRNA (scramble) and treated with cisplatin or control normal saline (N.S) (*n* = 5 for each group). Student’s two-tailed t-test, *P < 0.05; ** *P* < 0.01; ns, no significance. Error bars, mean ± SD

The lentivirus-mediated shRNAs specifically targeting LINC00173 were introduced to establish LINC00173 stable silenced cells A549-shLINC00173 and PC9-shLINC00173 so as to confirm the role of LINC00173 in DDP chemoresistance (Supplementary Fig. 2a). Subsequently, plate cloning, cell viability, and IC_50_ value experiments were conducted after treatment with DDP. The results indicated that colony formation, cell growth and cell viability were enhanced in LINC00173-deleted LUAD cells in the presence of DDP (Fig. 2a, 2b and 2d). Meanwhile, LINC00173 downregulation restrained the chemosensitivity of LUAD cells, with a significant increase in the IC_50_ value (Fig. 2c). However, the aforementioned results were not obtained in the absence of DDP (Supplementary Fig. 2b and 2c). Correspondingly, LINC00173 overexpression in DDP-resistant A549-DDP and PC9-DDP cells was implemented to ensure the effect of LINC00173 on DDP resistance. After the successful overexpression of LINC00173 (Supplementary Fig. 3a), IC_50_ value, cell viability, and colony formation of DDP-resistant cell lines sharply decreased in the presence of DDP (Supplementary Fig. 3b, 3c, and 3d, respectively), but the percentage of apoptotic cells notably increased (Supplementary Fig. 3e), implying the rescued chemosensitivity to DDP by LINC00173 transfection. These results suggested that LINC00173 was possibly involved in LUAD cell sensitization to DDP. LINC00173-silenced cells (A549-sh1/2 and PC9-sh1/2) or control shRNA cells (Scramble) were labeled with GFP as a transfection indicator to determine whether LINC00173 affected the sensitivity of LUAD cells to DDP, while their parental A549 and PC9 cells were labeled with DsRed. An equal number of GFP-labeled LINC00173 shRNA1/2 transfected cells and DsRed-labeled parental cells were then mixed and treated with DDP for 48 h. After incubation with DDP, the proportion of DsRed-labeled cells significantly reduced and that of GFP-labeled cells was elevated in the LINC00173 depletion group, whereas neither the proportion of DsRed-labeled cells nor that of GFP-labeled cells changed in the control shRNA-transfected group (Scramble) (Fig. 2e). This detection further verified that LINC00173 depletion markedly augmented LUAD resistance to DDP. We performed cell apoptosis assays with flow cytometry and detected hallmark proteins in the apoptotic pathway with immunoblotting to confirm that the chemoresistance to DDP caused by LINC00173 depletion was due to the inactivation of apoptosis. Apoptosis assays were performed using the Smart Silencer to transiently repress the expression of LINC00173 (Supplementary Fig. 2d). As shown in Fig. 2f and 2g, the apoptotic cells and apoptotic protein markers PARP, cleaved-PARP (c-PARP), caspase-3, and cleaved-caspase3 (c-CASP3) notably diminished in the LINC00173 knockdown group compared with the control group, whereas the critical apoptosis suppressor BCL2 expression was elevated after LINC00173 depletion when treated with DDP. Nevertheless, in the absence of DDP, no significant difference was found between LINC00173-depleted cells and control LUAD cells in apoptosis assays and apoptosis-related protein detection (Supplementary Fig. 2e and 2f). An in vivo investigation was then conducted by subcutaneously inoculating LINC00173-depleted and control A549 LUAD cells into the flank of nude mice. After DDP treatment every other day for 2 weeks, tumors of A549-shLINC00173-1 were markedly larger than those of A549 cells with scrambled shRNA (Fig. 2h–2j), which further affirmed the effect of LINC00173 on LUAD chemotherapeutic sensitivity to DDP. Besides, tumors in both LINC00173-silenced and control groups after N.S injection showed no significant difference (Fig. 2h–2j). The aforementioned results demonstrated that, in the presence of DDP, LINC00173 depletion conspicuously triggered the chemoresistance of LUAD cells to DDP by inactivating cell apoptosis, suggesting that LINC00173 might be a key factor in acquired chemoresistance in LUAD.

### LINC00173 promoted PROCA1 expression by acting as a competing endogenous RNA for miR-1275

**Fig. 3:**
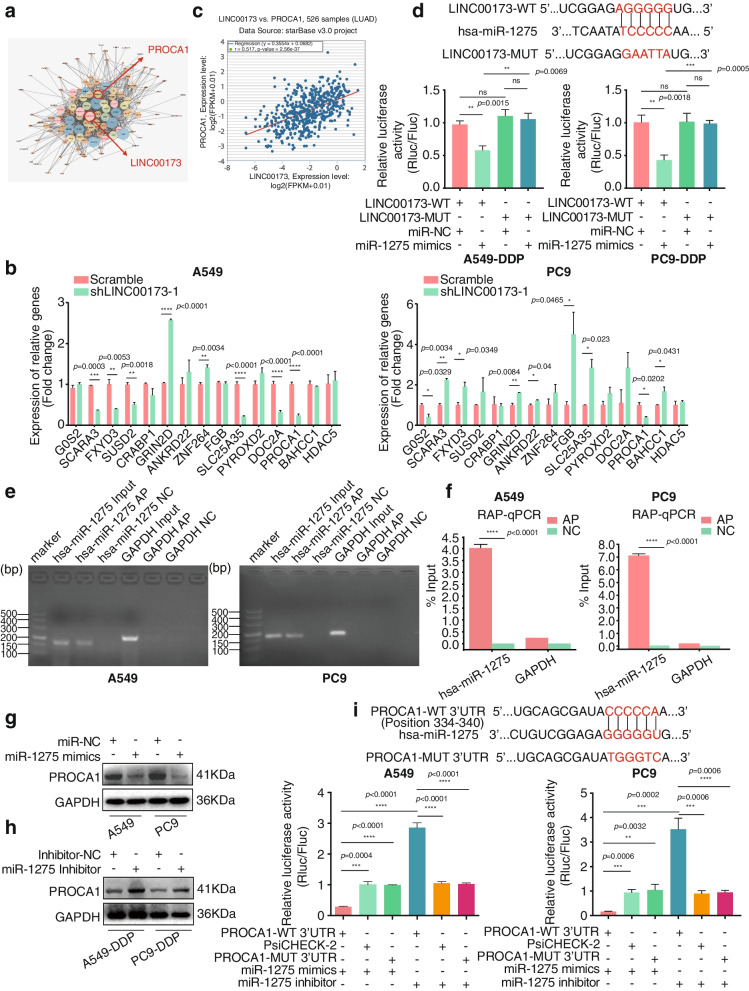
LINC00173 acts as a competing endogenous RNA to regulate PROCA1 by sponging miR-1275. a. Weighted gene co-expression network analysis (WGCNA) showed the closely relevant genes with LINC00173, including PROCA1. b. The effect of stable knockdown of LINC00173 on the expression level of candidate target genes verified by qRT-PCR in A549 and PC9 cells. Student’s two-tailed t-test, *P < 0.05; **P < 0.01; ***P < 0.001; ****P < 0.0001. c. The correlation between LINC00173 and candidate gene PROCA1 by bioinformatics analysis (starBase_v3.0). d. miR-1275 mimics or negative control (NC) was co-transfected with LINC00173-WT or MUT in A549-DDP/PC9-DDP cells. Luciferase activity was measured by luciferase assay. Student’s two-tailed t-test, **P < 0.01; ***P < 0.001; ns, no significance. e and f. RNA antisense purification (RAP) assay and agarose gel electrophoresis confirmed the binding of miR-1275 with LINC00173 in A549/PC9 cells. Student’s two-tailed t-test, ****P < 0.0001. g and h. The effect of miR-1275 on the protein expression of PROCA1 was verified by Western blot. GAPDH was used as a loading control. i. Luciferase reporter assay was used to determine miR-1275 directly targets the 3′-UTR of PROCA1. Student’s two-tailed t-test, **P < 0.01; ***P < 0.001; ****P < 0.0001. Error bars, mean ± SD

Weighted gene co-expression network analysis (WGCNA) was applied to screen genes closely related to LINC00173 (Fig. 3a) further to study the specific mechanism of action of LINC00173. Fifteen candidates were found to display significant differential expression. QRT-PCR analyses verified the alteration of their expression after LINC00173 depletion in A549 and PC9 cells. Of the 15 candidate genes, only GRIN2D and PROCA1 in both A549 and PC9 cells exhibited consistent variation in expression after LINC00173 depletion, among which PROCA1 expression positively correlated with LINC00173 expression (Fig. 3b). PROCA1 caught our attention because the correlation index between LINC00173 and PROCA1 was as high as 0.517 (Fig. 3c) when we compared the expression correlation between LINC00173 and the aforementioned candidates in the LUAD TCGA database. Besides, bioinformatics analysis in the same RNA-seq data (GSE43493) with the LINC00173 screening data set showed that PROCA1 was downregulated in LUAD DDP-resistant A549-DDP cells, which was consistent with the downregulation of LINC00173 (Supplementary Fig. 4a). The ISH assay described in Fig. 1 exhibited that LINC00173 was mainly expressed in the cytoplasm of LUAD (Fig. 1d). As cytoplasmic lncRNAs frequently function as competing endogenous RNAs (ceRNAs) to regulate downstream mRNAs by sponging miRNAs [6, 28, 29], the potential activity of LINC00173 as ceRNA in LUAD was explored. The DIANA database (http://carolina.imis.athenainnovation.gr/diana_tools/web/index.php) was used to screen miRNAs interacting with LINC00173, and miR-1275 was found as a predicted target. QRT-PCR analyses revealed that endogenous miR-1275 expression level in DDP-resistant LUAD cells could be significantly depressed by LINC00173 overexpression and restored with the addition of miR-1275 mimics (Supplementary Fig. 4b). The luciferase reporter assay revealed that miR-1275 mimics significantly inhibited the luciferase reporter activity of LINC00173, but not of mutant LINC00173 in A549-DDP and PC9-DDP cells (Fig. 3d). The RAP assay further verified the direct binding of miR-1275 with LINC00173 in A549/PC9 LUAD cells (Fig. 3e and 3f). The endogenous mRNA expression level of miR-1275 and PROCA1 was measured in DDP-resistant and their parental LUAD cells to further investigate the correlation between miR-1275 and PROCA1. The data showed that miR-1275 expression level remarkably increased in DDP-resistant LUAD cells, whereas PROCA1 expression reduced compared with that in the corresponding parental cells (Supplementary Fig. 4c and 4d), implying that miR-1275 expression was negatively associated with PROCA1 and both of them might take part in the resistance of LUAD cells to DDP. Interestingly, when analyzing the target genes of miR-1275 with TargetScan, PROCA1 was found as a potential target of miR-1275. The qRT-PCR and Western blot assays were, respectively, performed to assess whether miR-1275 could suppress the expression of PROCA1 at the mRNA and protein levels. The results indicated that miR-1275 mimics downregulated PROCA1 mRNA and protein expression in A549 and PC9 cells (Supplementary Fig. 4e and Fig. 3g), while miR-1275 inhibitors upregulated PROCA1 at both mRNA and protein levels in DDP-resistant LUAD A549-DDP and PC9-DDP cells (Supplementary Fig. 4f and Fig. 3h). We constructed luciferase reporters with WT (psiCHECK-2/PROCA1-wt-PROCA1 3′-UTR) and MUT of PROCA1 (psiCHECK-2/PROCA1-mut-PROCA1 3′-UTR) to further confirm that miR-1275 directly bound to the 3′-UTR of PROCA1. The WT or MUT of the PROCA1 vector was, respectively, co-transfected into A549 and PC9 cells with miR-1275 mimics or inhibitors. As shown in Fig. 3i, the upregulation of miR-1275 by its mimics diminished the luciferase activity, while the downregulation of miR-1275 by its inhibitors augmented the luciferase activity compared with either mutant or empty vector controls, indicating that PROCA1 was a downstream target of miR-1275. Collectively, these findings suggested that LINC00173 functioned as a ceRNA to positively regulate PROCA1 by sponging miR-1275 in LUAD.

### LINC00173 attenuated DDP resistance in LUAD by mediating the miR-1275/PROCA1 axis

**Fig. 4:**
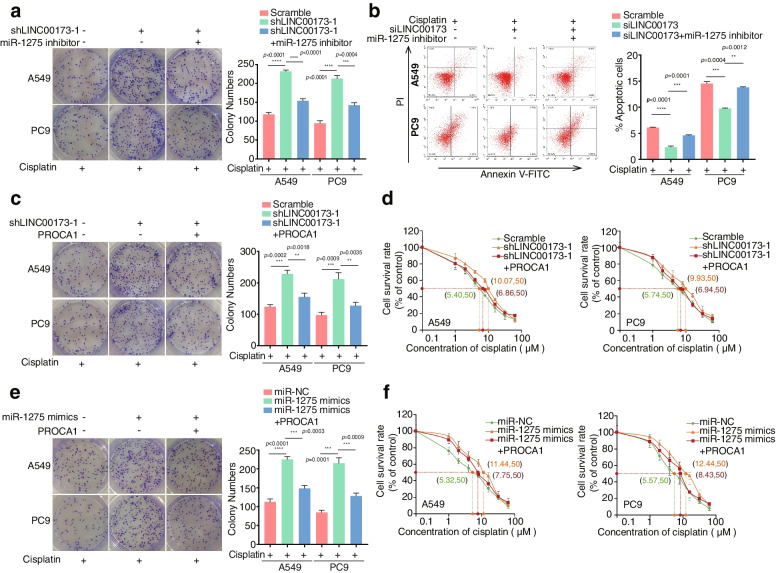
miR-1275/PROCA1 axis is essential for LINC00173-induced chemosensitivity to DDP in LUAD. a. miR-1275 inhibitors or negative control was transfected into LINC00173 stably silenced cells and cultured with cisplatin. Colony formation assay was performed to determine the effect of miR-1275 inhibition on the colony number of LINC00173 downregulated A549 and PC9 cells. Student’s two-tailed t-test, ***P < 0.001, ****P < 0.0001. b. miR-1275 inhibitors or negative control was co-transfected with LINC00173 smart silencer and cultured with cisplatin. Apoptosis of A549 and PC9 cells were measured by flow cytometry. Student’s two-tailed t-test, **P < 0.01; ***P < 0.001; ****P<0.0001. c-d. Overexpression of PROCA1 inhibited the chemoresistance of A549-shLINC00173-1 and PC9-shLINC00173-1 cells to cisplatin as examined by colony formation assay (c) and IC_50_ value (d). Student’s two-tailed t-test, **P < 0.01; ***P < 0.001. e–f. Co-transfection of PROCA1 with miR-1275 mimics inhibited the chemoresistance of A549 and PC9 cells to cisplatin as measured by colony formation assay (e) and IC_50_ value (f). Student’s two-tailed t-test, ***P < 0.001, ****P < 0.0001. Error bars, mean ± SD

Based on the aforementioned data, we preliminarily hypothesized that LINC00173 directly bound with miR-1275 to abrogate its inhibition of PROCA1. As shown in Supplementary Fig. 4c and 4d, we verified that miR-1275 and PROCA1 might be involved in the resistance of LUAD to DDP. Next, the role of PROCA1 and miR-1275 in LINC00173-mediated DDP chemoresistance was determined in LUAD. Functional detections further revealed that the IC_50_ value to DDP was markedly elevated in miR-1275 mimics-transducted LUAD cells (Supplementary Fig. 4 g), while it was reduced in DDP-resistant LUAD cells transducted with miR-1275 inhibitor (Supplementary Fig. 4 h). Subsequently, a series of retrieval assays were designed to identify the effect of PROCA1 and miR-1275 on LINC00173-mediated DDP resistance. First, miR-1275 inhibitors were transducted into LINC00173-depleted cells and treated with DDP. It was found that the repression of miR-1275 abolished the promotion of IC_50_ value, cell viability, colony formation, and apoptotic inhibition induced by LINC00173 depletion in the presence of DDP (Supplementary Fig. 5a and 5b, Fig. 4a and 4b). Moreover, Western blot analyses showed a change in the apoptotic protein levels, including the enhanced c-CASP3 and c-PARP, and the concomitant reduced expression of anti-apoptotic protein BCL2, with the addition of miR-1275 inhibitor (Supplementary Fig. 5c). These findings indicated that LINC00173 accelerated apoptosis to enhance the sensitivity of LUAD cells to DDP by inhibiting miR-1275. Next, similar results were obtained when PROCA1 was introduced into LINC00173-silenced LUAD cells, where PROCA1 reversed the stimulative effect of LINC00173 depletion on colony formation (Fig. 4c), IC_50_ value (Fig. 4d), cell viability (Supplementary Fig. 5d), and apoptotic inhibition (Supplementary Fig. 5e) in the presence of DDP. Furthermore, PROCA1 was co-transfected with miR-1275 mimics to identify their regulatory relationship in LUAD resistance to DDP. We discovered that the addition of PROCA1 notably abrogated the promoting effect of miR-1275 on the colony formation (Fig. 4e), IC_50_ value (Fig. 4f), and cell viability in the presence of DDP (Supplementary Fig. 5f). Together, LINC00173 abrogated chemotherapeutic resistance by acting as a ceRNA to bind to miR-1275, and thus abolished its transcriptional repression of PROCA1 in LUAD.

### PROCA1 interacted with ZFP36L2 to reduce BCL2 mRNA stability and activate the mitochondrial apoptosis pathway

**Fig. 5:**
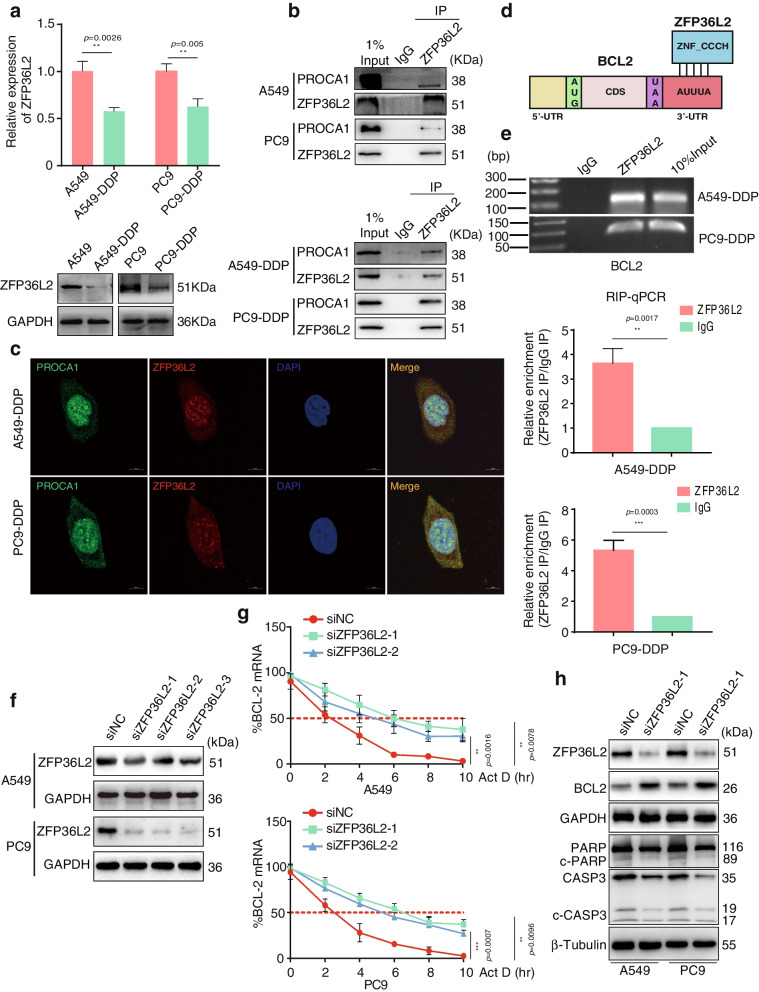
PROCA1 interacts with ZFP36L2 to decrease BCL2 mRNA stability and activate the mitochondrial apoptosis pathway. a. QRT-PCR (upper) and Western blot (lower) assays showing the expression level of ZFP36L2 in A549-DDP and PC9-DDP cells and their parental cell lines. Student’s two-tailed t-test, **P < 0.01. b. Co-IP experiments proved the interaction of endogenous ZFP36L2 and PROCA1 in A549/PC9 and A549-DDP/PC9-DDP cells. c. Co-localization of ZFP36L2 and PROCA1 in A549-DDP and PC9-DDP was detected by confocal immunofluorescence microscopy (scale bar, 10 μm). d. Schematic of ZFP36L2 binding to the 3' untranslated region of BCL2. e. RIP assay and agarose gel electrophoresis were used to corroborate the binding of ZFP36L2 protein to BCL2 mRNA. Student’s two-tailed t-test, **P < 0.01; ***P < 0.001. f. The interference efficiency of siRNA to ZFP36L2 was detected by western blot assays. GAPDH was used as a loading control. g. After ACT-D (1 μg/ml) treatment, BCL2 mRNA degradation was monitored by qRT-PCR in A549/PC9-siZFP36L2 or A549/PC9-control cells. Student’s two-tailed t-test, **P < 0.01; ***P < 0.001. h. Apoptosis-related proteins, including PARP, cleaved-PARP (c-PARP), Caspase3, cleaved-Caspase3 (c-CASP3) and BCL2 were analysed by western blot assays in ZFP36L2 inhibited A549 and PC9 cells. GAPDH and β-Tubulin were used as loading controls. Error bars, mean ± SD

To illustrate the regulatory mechanism of PROCA1 participating in LUAD apoptosis and chemotherapeutic resistance, BioGRID (https://thebiogrid.org/) online analysis was utilized to identify PROCA1 interacting proteins. ZFP36L2 was screened as a candidate, and then we preliminarily identified using qRT-PCR and Western blot that ZFP36L2 expression significantly decreased in A549-DDP and PC9-DDP cells compared with their control cells (Fig. 5a), implying a role for ZFP36L2 in LUAD chemoresistance to DDP. Endogenous Co-IP confirmed that PROCA1 interacted with ZFP36L2 in both A549/PC9 and A549-DDP/PC9-DDP cells (Fig. 5b). Next, the co-localization of PROCA1 and ZFP36L2 in A549-DDP/PC9-DDP cytoplasm was observed by immunofluorescence confocal microscopy (Fig. 5c), further visually confirming their interaction. ZFP36L2 was previously reported as a member of the ZFP36 family and was an RNA-binding protein (RBP) containing CCCH zinc-finger structure interacting with Au-rich elements (AUUUA sequence) in the 3'-UTR of mRNA to accelerate the degradation of mRNA or inhibit protein translation [30, 31]. Fortunately, a previous study demonstrated an AUUUA sequence in the BCL2 mRNA sequence [32]. Therefore, we hypothesized that ZFP36L2 could bind to BCL2 mRNA, and then confirmed the existence of a putative binding site at the 3'-UTR of BCL2 (Fig. 5d). The RIP analysis further verified the binding of endogenous ZFP36L2 protein to endogenous BCL2 mRNA (Fig. 5e). Based on these results, we then sought to identify the effect of ZFP36L2 on BCL2 mRNA stability. Three independent siRNAs targeting ZFP36L2 were designed to downregulate the expression of ZFP36L2, among which the two with higher interfering efficiency were chosen to perform the subsequent assays (Fig. 5f). The actinomycin D (Act-D) treatment was applied to assess the stability of BCL2 mRNA. After the inhibition of ZFP36L2, the cells were incubated with Act-D (1 μg/mL) and collected to detect BCL2 expression after 0, 2, 4, 6, 8, and 10 h. BCL2 mRNA degradation was monitored by qRT-PCR analyses. As shown in Fig. 5g, the half-life of BCL2 mRNA was approximately 2 h in control cells, while the degradation of BCL2 mRNA was significantly delayed in ZFP36L2-depleted cells, suggesting that ZFP36L2 downregulation contributed to BCL2 stabilization. BCL2 is well known as a key anti-apoptotic gene involved in inhibiting the mitochondrial apoptosis pathway [33, 34]. Therefore, we further explored the expression of several critical factors in this pathway. As expected, an increase in BCL2 and a reduction in PARP, c-PARP, Caspase3, and c-CASP3 were observed following ZFP36L2 depletion, indicating that the apoptotic pathway was inactivated (Fig. 5h). Taken together, these data demonstrated that PROCA1 recruited ZFP36L2 to impair the stability of BCL2 mRNA, thus activating the apoptotic pathway.

### PROCA1/ZFP36L2/BCL2 axis was essential for LINC00173-mediated chemosensitivity to DDP in LUAD

**Fig. 6:**
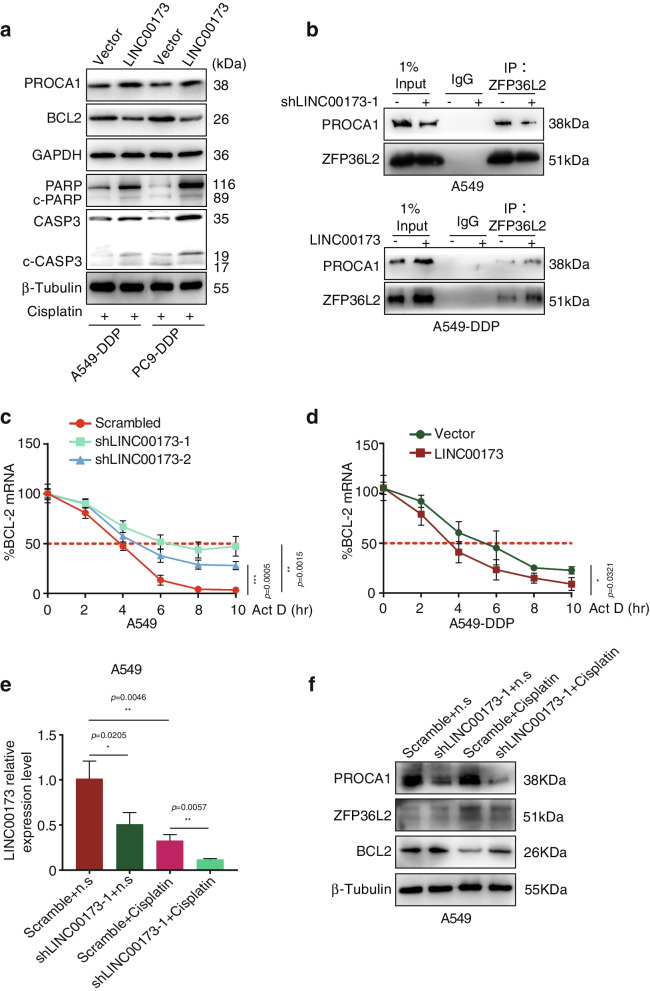
LINC00173 mediated chemosensitivity to DDP in LUAD via PROCA1/ZFP36L2/BCL2 axis. a. The protein level of PROCA1, BCL-2, PARP, cleaved-PARP (c-PARP), Caspase3 and cleaved-Caspase3 (c-CASP3) in LINC00173 transfected A549-DDP and PC9-DDP cells (cultured with cisplatin) were examined by western blot. GAPDH and β-Tubulin were used as loading controls. b. Co-immunoprecipitation analysed the level of PROCA1 binding to ZFP36L2 in A549 and A549-DDP cells (cultured with cisplatin) transfected with shLINC00173 or LINC00173, respectively. c and d. BCL2 mRNA degradation was monitored by qRT-PCR in A549-shLINC00173 (c) or A549-DDP-LINC00173 cells cultured with cisplatin (d). e. The expression of LINC00173 in xenograft tumor tissues generated from A549 cells expressing either shRNAs targeting LINC00173 (shLINC00173-1) or nontargeting shRNA (scramble) and treated with cisplatin or control normal saline (N.S) f. The protein levels of PROCA1, ZFP36L2, and BCL2 in xenograft tumor tissues generated from A549 cells expressing either shRNAs targeting LINC00173 (shLINC00173-1) or nontargeting shRNA (scramble) and treated with cisplatin or control normal saline (N.S). Student’s two-tailed t-test, *P < 0.05; **P < 0.01; ***P < 0.001. Error bars, mean ± SD

In the previous experiments, we elucidated that PROCA1 was crucial to improving the efficacy of DDP against LINC00173-depleted LUAD cells (Fig. 4c and 4d, and Supplementary Fig. 5d and 5e). In this section, we further confirmed in DDP-resistant cells that PROCA1 was upregulated after LINC00173 overexpression (Fig. 6a). The Co-IP assay was performed to identify the binding of PROCA1 with ZFP36L2 affected by LINC00173. The results showed that PROCA1 pulled down by ZFP36L2 was visibly reduced when LINC00173 was interfered in A549 cells in the presence of DDP. However, an opposite result was achieved in A549-DDP cells when LINC00173 was overexpressed (Fig. 6b), implying that the expression level of LINC00173 positively determined the recruitment of PROCA1 to ZFP36L2. Subsequently, BCL2 mRNA stability was measured by qRT-PCR at different time points after Act-D treatment. The half-life of BCL2 mRNA was extended when knocking down LINC00173 in A549 cells with DDP treatment (Fig. 6c). Nevertheless, the half-life of BCL2 mRNA was shortened in LINC00173-overexpressed A549-DDP cells (Fig. 6d), along with the diminished protein level of BCL2 and the augmented expression of downstream apoptotic proteins PARP, cleaved-PARP, caspase-3, and c-CASP3 (Fig. 6a). Collectively, LINC00173 sensitized LUAD cells to DDP by enhancing the binding of PROCA1 with ZFP36L2, which accelerated the degradation of BCL2 mRNA and further activated the mitochondrial apoptotic pathway. To further confirm the aforementioned finding, the expression of LINC00173, PROCA1, ZFP36L2, and BCL2 was subsequently assessed in the xenograft tumor tissues of control and LINC00173-deleted group with DDP or N.S treatment. The results revealed that LINC00173 expression was dramatically declined in LINC00173 knockdown groups (sh-LINC00173-1) compared with control groups (Scramble) irrespective of the cisplatin or N.S treatments (Fig. 6e). Moreover, the expression of PROCA1 significantly reduced after LINC00173 knockdown. Nevertheless, a marked change in ZFP36L2 was not observed between LINC00173 knockdown and control groups (Fig. 6f), probably because the protein expression of ZFP36L2 recruited by PROCA1 was decreased by reduced PROCA1 in the process of LINC00173 depletion–induced chemoresistance to DDP (Fig. 6b). However, the protein level of ZFP36L2 was not significantly changed by LINC00173. Additionally, BCL2 expression was notably elevated in the LINC00173 knockdown group compared with the control group only in the presence of DDP (Fig. 6f), which was in accordance with the previous in vitro results that LINC00173 knockdown in LUAD cells did not induce apoptosis or affect apoptosis-related proteins including BCL2 in the absence of DDP (Supplementary Fig. 2e and 2f), whereas LINC00173 knockdown did increased the expression of BCL2 and impeded cell apoptosis in the presence of DDP (Fig. 2f and 2e), thus triggering the chemoresistance of LUAD cells to DDP. Taken together, these data suggested that LINC00173 induced chemosensitivity to DDP in LUAD via the PROCA1/ZFP36L2/BCL2 axis.

### Low expression of LINC00173 in DDP-resistant LUAD was attributed to negative regulation by c-Myc

**Fig. 7:**
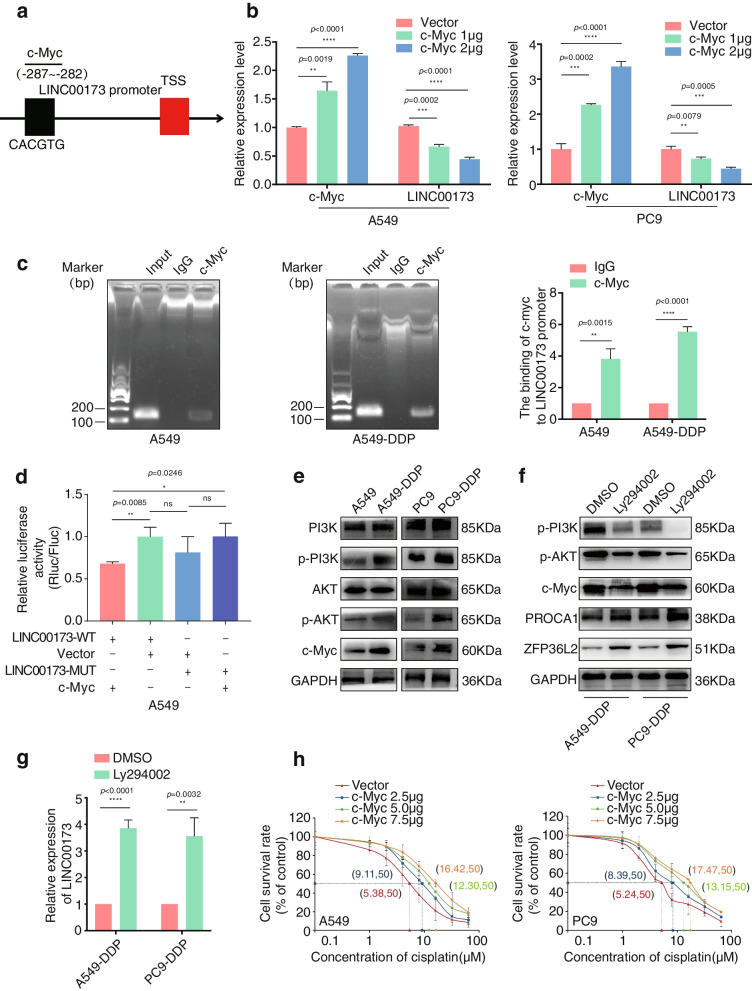
Low expression of LINC00173 in DDP-resistant LUAD attributes to the negative regulation of c-Myc. a. Predicted c-Myc binding site in the promoter region of LINC00173. b. Transfection of c-Myc plasmids suppressed LINC00173 expression in A549 and PC9 cells. Student’s two-tailed t-test, **P < 0.01; ***P < 0.001; ****P < 0.0001. c. ChIP assay and agarose gel electrophoresis verified the binding of c-Myc to LINC00173 promoter in A549 and A549-DDP cells. Student’s two-tailed t-test, **P < 0.01; ****P < 0.0001. d. Luciferase reporter assay further validated c-Myc was directly binding to LINC00173 promoter. Student’s two-tailed t-test, *P < 0.05; **P < 0.01; ns, no significance. e. Western blot analysis of PI3K, p-PI3K, AKT, p-AKT and c-Myc expression in A549 and A549-DDP cells. GAPDH was used as a loading control. f. Western blot detection of p-PI3K, p-AKT, c-Myc, PROCA1 and ZFP36L2 expression in A549-DDP and PC9-DDP cells after treated with PI3K inhibitor LY294002. GAPDH was used as a loading control. g. The expression level of LINC00173 was obviously increased in A549-DDP and PC9-DDP cell lines after treated with LY294002. h. The IC_50_ values to cisplatin were detected in A549 and PC9 cells transfected with a gradient concentration of c-Myc plasmids. Student’s two-tailed t-test, **P < 0.01; ****P < 0.0001. Error bars, mean ± SD

The aforementioned results demonstrated that the LINC00173 expression was downregulated in DDP-resistant LUAD and its low expression contributed to the chemotherapeutic resistance of LUAD. To clarify the specific mechanism of LINC00173 downregulation in the process of DDP-based resistance of LUAD, we explored the upstream transcriptional regulation of LINC00173 in LUAD cells. The PROMO online algorithms were used to analyze the 2-kb sequence upstream from the transcription start site of LINC00173. A potential c-Myc-binding site was predicted at − 287 to − 282 in the putative promoter of LINC00173 (Fig. 7a). Accordingly, c-Myc plasmids were introduced into A549 and PC9 cells with a gradient concentration, and their effect on the LINC00173 expression was evaluated by qRT-PCR. As shown in Fig. 7b, the LINC00173 expression level negatively correlated with c-Myc concentration, suggesting that c-Myc might act as an upstream suppressor of LINC00173. The ChIP assay was carried out to confirm the binding of c-Myc to the LINC00173 promoter in both A549 and A549-DDP-resistant cells (Fig. 7c). Consistently, diminished luciferase activity of the LINC00173 promoter was observed in the c-Myc-overexpressed LUAD cells, whereas the luciferase activity was not significantly influenced by c-Myc when the putative binding site was mutated (Fig. 7d). Therefore, the aforementioned results demonstrated that c-Myc bound to the promoter of LINC00173 and inhibited the transcription of LINC00173 in LUAD. Previous reports have fully revealed that the PI3K/Akt signal was overactivated in the process of DDP resistance [27, 35], and c-Myc was positively regulated by PI3K/Akt [36]. We hypothesized that the LINC00173 expression was suppressed by the DDP-activated PI3K/Akt/c-Myc signal in LUAD. To verify our hypothesis, we detected the expression using Western blot analysis and found significant elevation of p-PI3K, p-Akt, and c-Myc protein levels in DDP-resistant LUAD cells compared with the corresponding parental cells (Fig. 7e). Subsequently, LY294002, a specific PI3K inhibitor, was applied to block the PI3K/Akt pathway in the DDP-resistant LUAD cells. The Western blot and qRT-PCR analyses exhibited that LY294002 markedly attenuated the expression of p-PI3K, p-Akt, and c-Myc but restored the expression of LINC00173 and its downstream signal molecules PROCA1 and ZFP36L2 (Fig. 7f and 7g). Moreover, the effect of c-Myc on DDP sensitivity was examined by transfecting A549 and PC9 cells with a gradient concentration of c-Myc plasmids for 48 h. The cells were then treated with a gradient concentration of DDP to detect the IC_50_ value in each group. The results revealed that the IC_50_ value was markedly elevated in c-Myc over-expressed A549 and PC9 cells compared with the cells transfected with vector (negative control). Also, the IC_50_ value increased in a dose-dependent manner, suggesting that c-Myc promoted the resistance to DDP in LUAD (Fig. 7h). In summary, these results revealed that activated PI3K/AKT/c-Myc signal in DDP-resistant LUAD cells contributed to the transcriptional inhibition of LINC00173.

## Discussion

Acquired drug resistance to cisplatin (DDP)-based chemotherapeutics has been proven as a considerable cause of relapse and, thus, poor prognosis of patients with LUAD. As a member of the first-line standard chemotherapy drug, DDP plays a considerable role in the LUAD treatment [37]. The exploration of the molecular mechanism of DDP resistance in LUAD is therefore of great value to improvement in chemotherapy efficacy. Previous studies showed that LINC00173 acted as a tumor suppressor to inhibit cell proliferation and migration and induce apoptosis in NSCLC (including LUSC and LUAD) [18]. However, recent studies demonstrated that its upregulation in SCLC and LUSC promoted DDP chemotherapy resistance as a candidate oncogene [21, 22]. Lung cancer is broadly classified into two groups based on histology: (1) SCLC and (2) NSCLC primarily categorized into LUAD, LUSC, and large cell carcinoma [38]. SCLC has been suggested to arise from the pluripotent epithelial cells with neuroendocrine differentiation potential in the lung [39, 40]. Although LUSC and LUAD both belong to NSCLC, LUSC, as well as SCLC, generally arises in the more proximal airways, whereas LUAD originates in the peripheral airways such as alveolar type 2 cells [41, 42]. These studies suggested that LINC00173 played contrasting roles in LUAD compared with its roles in SCLC or LUSC. However, the role and molecular basis of LINC00173 in LUAD resistance to DDP are still undetermined.

In this study, we first downloaded the high-throughput mRNA expression data and screened the differentially expressed lncRNAs between A549 and its DDP-resistant strain A549-DDP. Interestingly, LINC00173 was significantly downregulated in A549-DDP cells, which was subsequently verified by qRT-PCR in chemoresistant LUAD cell lines A549-DDP and PC9-DDP. Furthermore, the clinical sample analysis indicated that patients with LUAD with lower expression of LINC00173 had worse chemotherapeutic responses and poorer overall survival. These data supported that the downregulated expression of LINC00173 was a significant event for DDP resistance in LUAD.

We first investigated the molecular basis of DDP to suppress LINC00173 in LUAD to further determine whether LINC00173 was involved in DDP resistance. C-Myc was identified as a key oncogenic transcription factor [43–46] binding to the promoter of LINC00173 and negatively modulating its expression in DDP-resistant LUAD cells. As the PI3K/Akt signal significantly correlated with DDP resistance [47–50] and c-Myc was a positive downstream regulator of PI3K/Akt [36], we speculated that DDP might repress LINC00173 expression by activating PI3K/Akt/c-Myc. Consistent with this hypothesis, PI3K-inhibitor LY294002 used in DDP-resistant cell lines successfully impaired the activity of PI3K/Akt/c-Myc signal, restoring the expression of LINC00173 in LUAD.

Acquired DDP resistance likely occurs due to complex reasons, including increased drug efflux [51], increased repair of damaged DNA [52], increased activation of pro-survival pathways, or inhibition of apoptotic pathways, which require the involvement of multiple genes and their products [53]. Thereinto, apoptosis inhibition is considered as one of the key mechanisms for chemotherapy resistance [54–58].To further explore whether downregulated LINC00173 was involved in DDP-acquired resistance in LUAD, we silenced LINC00173 in LUAD cells but did not observe a change in colony formation, cell viability, or apoptosis induction in the absence of DDP. These findings were contrary to the results obtained by Yang showing LINC00173 as a tumor suppressor in NSCLC [18]. However, the reason for this difference is unknown. Interestingly, with DDP treatment, LINC00173 depletion in LUAD cells significantly elevated the IC_50_ value, colony formation ability, and cell viability and restrained cell apoptosis. This was further verified in DDP-resistant LUAD cells, where LINC00173 overexpression led to a dramatic reduction in the IC_50_ value, colony formation, and viability in the presence of DDP. More importantly, the inhibition of LINC00173 by shRNAs markedly abrogated the therapeutic sensitivity of LUAD cells to DDP in vivo. Further, preliminary molecular experiments showed that LINC00173 suppressed the expression of anti-apoptotic key factor BCL2 [59] and induced the expression of apoptosis biomarkers c-CASP3 and c-PARP [60, 61].

These data demonstrated that although LINC00173 had no effect on cell survival without DDP treatment, it significantly promoted DDP-induced cell apoptosis and thus resulted in chemosensitivity to DDP in LUAD. These findings were markedly contrary to the role of LINC00173 in SCLC and LUSC, indicating a diversity in the drug resistance function of LINC00173 in different histologic lung cancer types [21, 22].

Previous studies reported that LINC00173 acted as a tumor suppressor by inhibiting the miR-182-5p-regulated AGER/NF-κB pathway to restrain cell proliferation and migration in NSCLC [18]. In this study, we used online prediction tools to search for the downstream ceRNA of LINC00173, and found miR-1275 as its candidate target. Subsequently, we first confirmed that miR-1275 expression increased in DDP-resistant LUAD cells and then proved its function in LUAD chemotherapeutic resistance to DDP. Consistent with previous findings, miR-1275 acted as an oncogene to promote LUAD progression [62].

WGCNA is a systematic biological method used to describe gene association patterns. It was used to screen the potential downstream target genes regulated by LINC00173 in the process of LUAD chemoresistance. PROCA1 was found to be positively related to LINC00173. So far, it was only reported to interact with Cyclin A1–CDK2 complex and participate in the occurrence and development of germ cell carcinoma [63]. Further predictions implied that LINC00173 might bind to miR-1275, which was just predicted to target PROCA1. Subsequent investigation confirmed that LINC00173 acted as a ceRNA to regulate downstream PROCA1 by sponging miR-1275 so as to induce cell apoptosis and chemosensitivity to DDP in LUAD.

To deeply explore the mechanism of PROCA1-mediated LINC00173/miR-1275 signal in the DDP chemosensitivity of LUAD, we used the BioGRID database to screen the potential interacting proteins of PROCA1. ZFP36L2 was considered a candidate target. ZFP36L2 is a member of the ZFP36 RNA-binding proteins (RBP) family, which contains a CCCH zinc-finger structure to bind to the AU-rich elements (AREs) in the 3'-UTR of mRNAs to accelerate mRNA degradation and inhibit protein translation [30, 31]. In recent studies, ZFP36L2 was reported as a target of AML1 to induce the apoptosis of leukemia cells, suggesting its inhibitory role in tumor pathogenesis [64]. In this study, we first determined the decreased expression of ZFP36L2 in DDP-acquired resistant LUAD cells and confirmed the interaction between ZFP36L2 and PROCA1 in A549 and A549-DDP cells, as well as the co-localization of both of them in the cytoplasm of A549-DDP cells. These data demonstrated that PROCA1 bound with ZFP36L2 in LUAD and was involved in LUAD-acquired resistance to DDP.

BCL2 is widely known as a vital apoptosis suppressor that promotes tumor pathogenesis and DDP chemotherapy resistance by blocking mitochondrial apoptosis signaling pathways, including suppressing c-CASP3 and c-PARP [65]. Intriguingly, the 3'-UTR of BCL2 contains AREs, which can be a binding site of ZFP36L2. Further studies confirmed that ZFP36L2 bound to the 3'-UTR of BCL2 mRNA to impair its stability and promote its degradation, ultimately inducing cell apoptosis and chemotherapeutic sensitivity to DDP via activating apoptosis signal in LUAD. Finally, LINC00173 transfection in DDP-resistant LUAD cells significantly restored the sensitivity of cells to DDP by regulating miR-1275/PROCA1/ZFP36L2 signal to expedite ARE-mediated BCL2 mRNA decay.

## Conclusions

This study was novel in demonstrating that the downregulation of LINC00173 was involved in the poor survival outcome of LUAD patients with DDP treatment. Further, LINC00173 inhibition was attributed to DDP-activated PI3K/Akt/c-Myc-induced transcriptional repression in the process of DDP-acquired chemotherapeutic resistance. Downregulated LINC00173 expression resulted in the reduced sponging of miR-1275 and thus suppressed the expression of PROCA1, which further diminished the recruitment of the RNA-binding protein ZFP36L2 to bind to the 3'-UTR of BCL2 mRNA and induce its degradation. This ultimately enhanced BCL2 mRNA stability, impeding cell apoptosis and promoting chemotherapeutic resistance to DDP in LUAD. The findings uniquely demonstrated an inhibitory role of LINC00173 in acquired DDP resistance in LUAD, which was inconsistent with its role in SCLC and LUSC. This study might provide an insight into the diverse roles of LINC00173 in different histologic types of lung cancer and provide a distinctive chemotherapeutic strategy for patients with LUAD differing from SCLC or LUSC.

## Supplementary Information

**Additional file 1. ** 

**Additional file 2.** 

**Additional file 3.**

**Additional file 4.**

**Additional file 5.** 

**Additional file 6.**

**Additional file 7.** 

**Additional file 8.**

**Additional file 9.**

## Data Availability

The datasets generated during and/or analyzed during the current study are available from the corresponding author upon reasonable request.

## Abbreviations

DDP: Cisplatin, *cis*-diamminedichloroplatinum
LUAD: Lung adenocarcinoma
NSCLC: Non-small-cell lung cancer
SCLC: Small-cell lung cancer
LUSC: Lung squamous cell carcinoma
LncRNA: Long noncoding RNA
ChIP: Chromatin immunoprecipitation
Co-IP: Co-immunoprecipitation
qRT-PCR: Quantitative real-time reverse transcription polymerase chain reaction
WGCNA: Weighted gene co-expression network analysis
RBP: RNA-binding protein
ARE: AU-rich element
ISH: In situ hybridization
