# Supplementary figures and images for: Downregulation of Linc00173 increases BCL2 mRNA stability via the miR-1275/PROCA1/ZFP36L2 axis and induces acquired cisplatin resistance of lung adenocarcinoma
Source: J Exp Clin Cancer Res. 2023 Jan 10;42:12. doi: 10.1186/s13046-022-02560-6 (PMC9830831; doi:10.1186/s13046-022-02560-6)

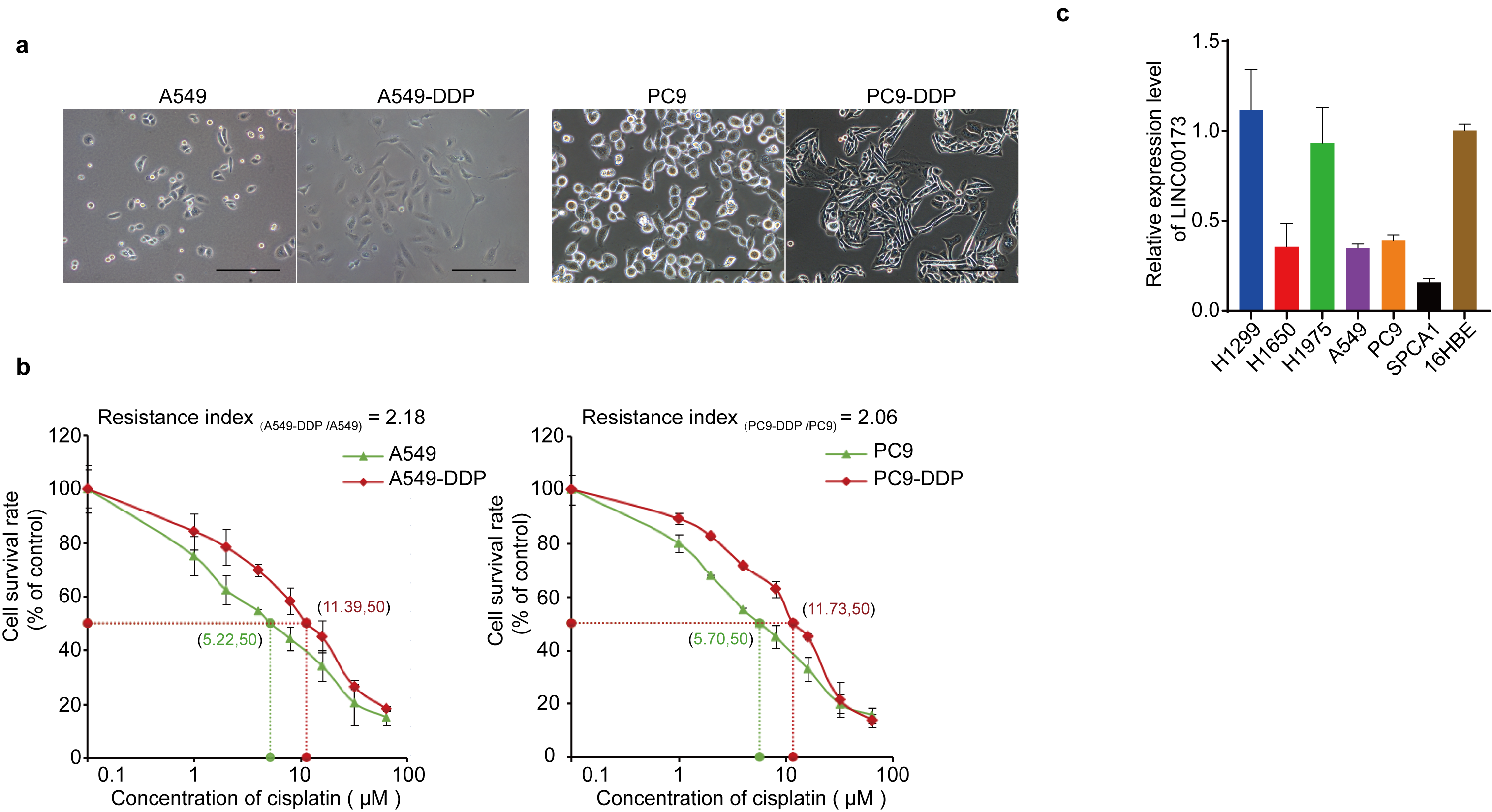

Supplement: Supplementary file 1 — Additional file 1. [file 13046_2022_2560_MOESM1_ESM.tif]

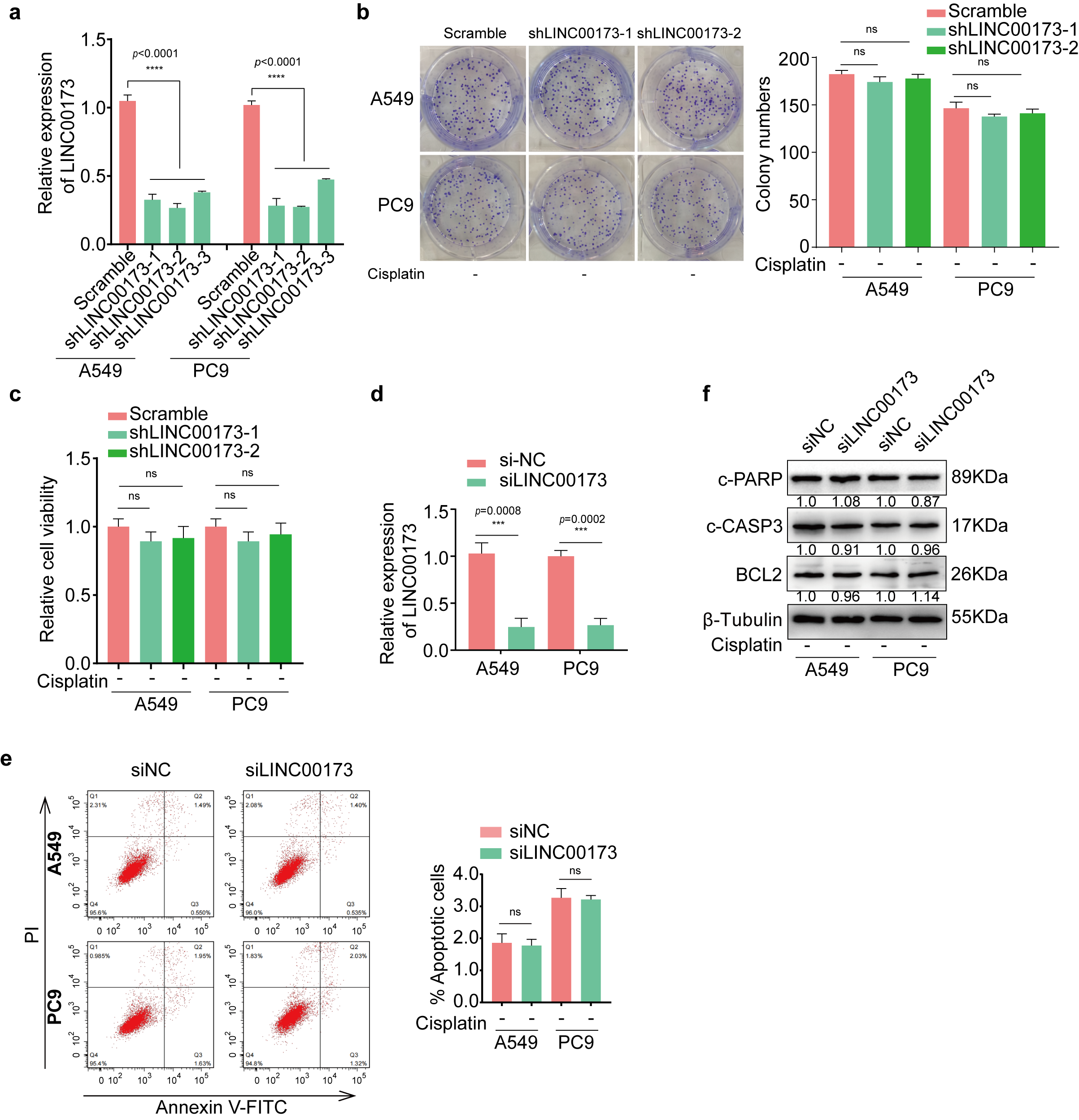

Supplement: Supplementary file 2 — Additional file 2. [file 13046_2022_2560_MOESM2_ESM.tif]

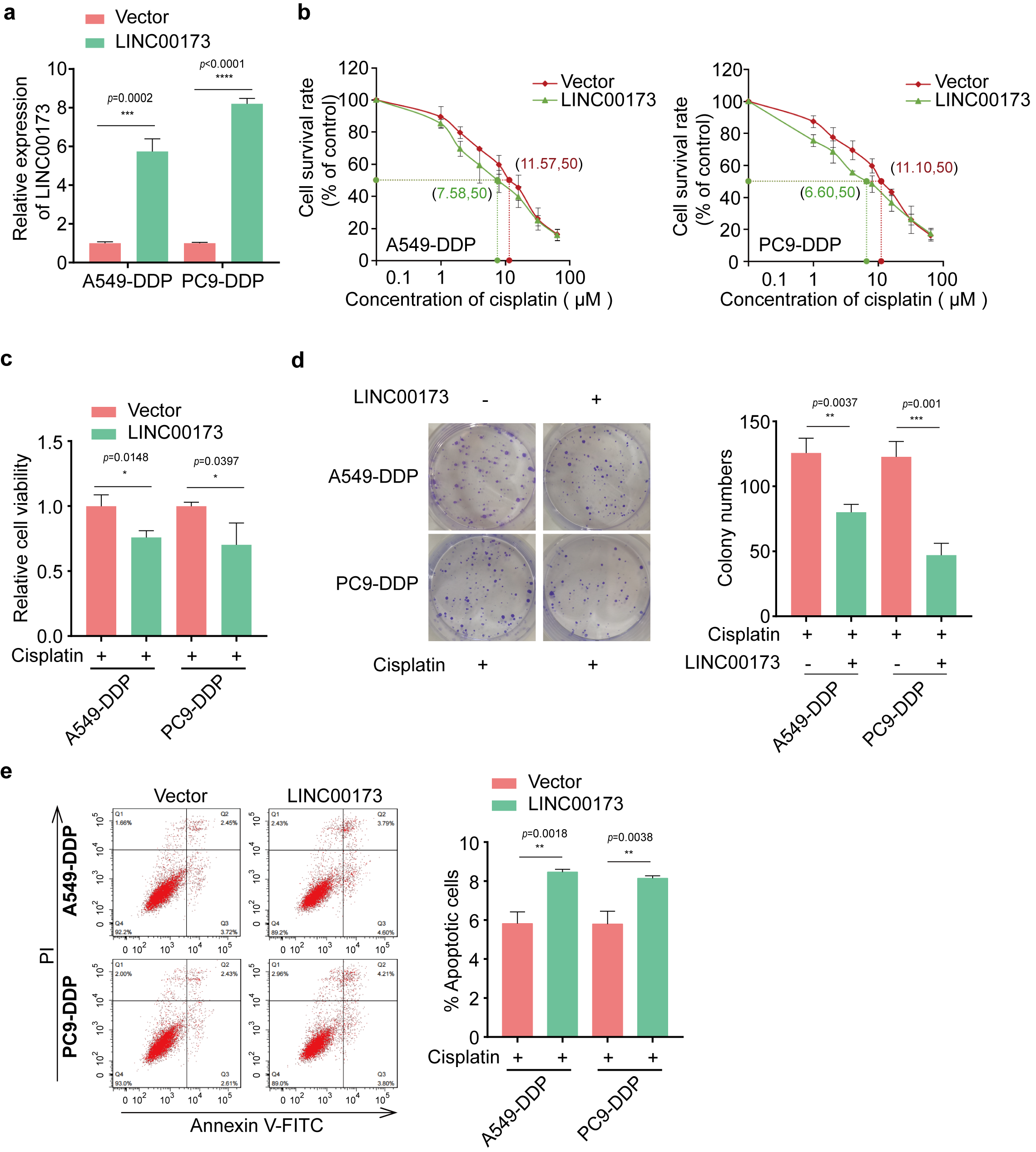

Supplement: Supplementary file 3 — Additional file 3. [file 13046_2022_2560_MOESM3_ESM.tif]

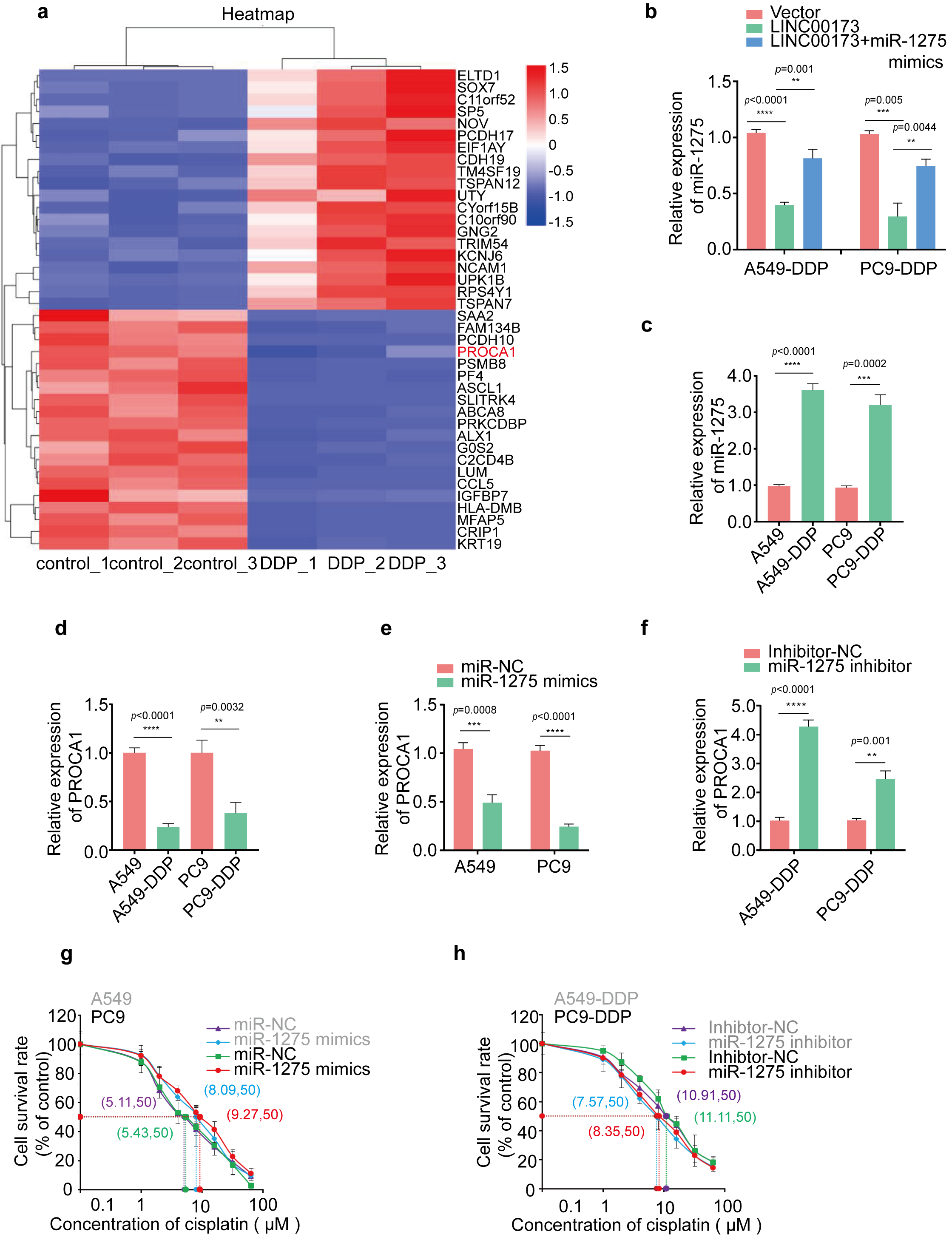

Supplement: Supplementary file 4 — Additional file 4. [file 13046_2022_2560_MOESM4_ESM.tif]

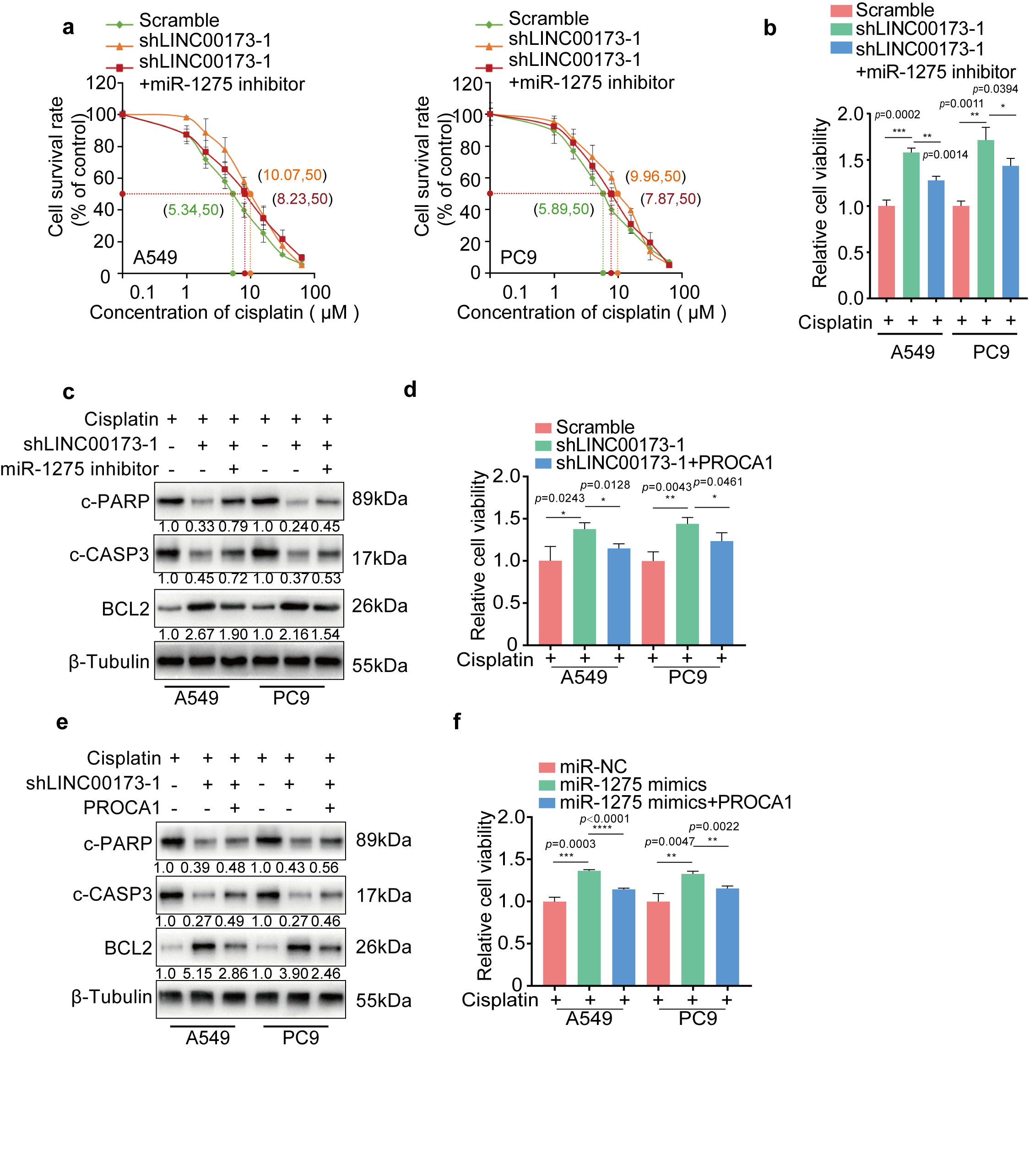

Supplement: Supplementary file 5 — Additional file 5. [file 13046_2022_2560_MOESM5_ESM.tif]
