# Supplementary material for: Downregulation of Linc00173 increases BCL2 mRNA stability via the miR-1275/PROCA1/ZFP36L2 axis and induces acquired cisplatin resistance of lung adenocarcinoma
Source: J Exp Clin Cancer Res. 2023 Jan 10;42:12. doi: 10.1186/s13046-022-02560-6 (PMC9830831; doi:10.1186/s13046-022-02560-6)
Supplement: Supplementary file 6 — Additional file 6. [file 13046_2022_2560_MOESM6_ESM.docx]

**Supplementary Table 1. The sequences of smart-silencer, shRNA, siRNA, miR-1275 mimics and miR-1275 inhibitor used in this study.**

| Product Name |  | Sequences |
| --- | --- | --- |
| RiboTM h-LINC00173  Smart Silencer |  | 5’-GGAATTCACACCCTATATT-3’  5’-GGCCCGCAGGAAATTTATA-3’  5’-GGAATCTCATTTGCCAAAT-3’  5’-GCTAAGTCCTGCACACGCTC-3’  5’-AAGTCCTGCACACGCTCCTC-3’  5’-CCGGCCCGCAGGAAATTTAT-3’ |
| shLINC00173-1 |  | 5’-TCAGAGGTGTTGATGAATGTT-3’ |
| shLINC00173-2 |  | 5’-TTGGAAGAGATGTGAATTGGA-3’ |
| shLINC00173-3 |  | 5’-GAGGCTCCCACCTGCTCTAAG-3’ |
| si- ZFP36L2-1 |  | 5’-ACCGCTCGTTTAGCGAGAA-3’ |
| si- ZFP36L2-2 |  | 5’-ACGTGCAAGTACGGCGAAA-3’ |
| si- ZFP36L2-3 |  | 5’-TGCCGCACCTTTCATACCA-3’ |

| miR-1275 mimics | Sense 5´-CCUCUGUGAGAAAGGGUGUG-3´  Antisense 3´-GGAGACACUCUUUCCCACAC-5´  Antisense 3´CAAGACAAUUGGGUAGGGGAGU5´ |
| --- | --- |
| Negative control | Sense 5´-UUCUCCGAACGUGUCACGUTT-3´  Antisense 3´-ACGUGACACGUUCGGAGAATT-5´ |
| miR-1275 inhibitor | 5´-CACACCCUUUCUCACAGAGG-3´ |
| Inhibitor negative control | 5´-UUUGUACUACACAAAAGUACUG-3´ |
