# Supplementary material for: Downregulation of Linc00173 increases BCL2 mRNA stability via the miR-1275/PROCA1/ZFP36L2 axis and induces acquired cisplatin resistance of lung adenocarcinoma
Source: J Exp Clin Cancer Res. 2023 Jan 10;42:12. doi: 10.1186/s13046-022-02560-6 (PMC9830831; doi:10.1186/s13046-022-02560-6)
Supplement: Supplementary file 7 — Additional file 7. [file 13046_2022_2560_MOESM7_ESM.docx]

**Supplementary Table 2. Correlation between the clinicopathological features and LINC00173 expression in LUAD.**

| **Clinicopathologic Factors** | **LINC00173 expression** | | ***p* Value** |
| --- | --- | --- | --- |
|  | **Low** | **High** |  |
| **Gender** |  |  |  |
| Male | 34(54.8%) | 28(45.2%) | 0.068 |
| Female | 26(38.8%) | 41(61.2%) |  |
| **Age (years)** |  |  |  |
| ≥60 | 26(41.3%) | 37(58.7%) | 0.244 |
| <60 | 34(51.5%) | 32(48.5%) |  |
| **T classification** |  |  |  |
| T1+T2 | 56(46.3%) | 65(53.7%) | 1.000 |
| T3+T4 | 4(50.0%) | 4(50.0%) |  |
| **N classification** |  |  |  |
| N0 | 32(45.1%) | 39(54.9%) | 0.717 |
| N1+N2+N3 | 28(48.3%) | 30(51.7%) |  |
| **Distant metastasis** |  |  |  |
| Negative | 42(42.0%) | 58(58.0%) | 0.056 |
| Positive | 18(62.1%) | 11(37.9%) |  |
| **pTNM stage** |  |  |  |
| I+II | 34(39.5%) | 52(60.5%) | **0.025** |
| III+IV | 26(60.5%) | 17(39.5%) |  |
