# Supplementary material for: Downregulation of Linc00173 increases BCL2 mRNA stability via the miR-1275/PROCA1/ZFP36L2 axis and induces acquired cisplatin resistance of lung adenocarcinoma
Source: J Exp Clin Cancer Res. 2023 Jan 10;42:12. doi: 10.1186/s13046-022-02560-6 (PMC9830831; doi:10.1186/s13046-022-02560-6)
Supplement: Supplementary file 8 — Additional file 8. [file 13046_2022_2560_MOESM8_ESM.docx]

**Supplementary Table 3. The primers used in this study.**

| Gene Names |  | Primers sequences (5’-3’) |
| --- | --- | --- |
| LINC00173 | Forward | CCAGCACAGCGTTCTTGGTCTC |
|  | Reverse | AGGTGCAGATCCAGGCGTACC |
| AL359062 | Forward | GGGAGTGCTGCTTACCATTTC |
|  | Reverse | AGCGGCTTGATCCCATAGTAG |
| NCRNA00230A | Forward | TAAGATGTCATCGGCGAGCG |
|  | Reverse | TTAAACAAGCCAGCCAAGCG |
| KRT19P3 | Forward | GGACCATCGAGTACCTGTGC |
|  | Reverse | AGCCAGACAGGCATTGTTGA |
| LOC100190940 | Forward | ACTGTGGTCGCTGAGAACTG |
|  | Reverse | GTTTCCGAGACCCACGTCAT |
| AK075442 | Forward | GCTCGATCAACTTTGCCAGT |
|  | Reverse | TCTCAGGGGGTTCACAGCAT |
| AB074163 | Forward | AAGAGGTGATCAGCCAAGCC |
|  | Reverse | ACGGAGCATGTGATCAAGCA |
| AL137603 | Forward | TTTGGTGATTGGCAGCCTCT |
|  | Reverse | AGGCTAAGAAGGCCTAACAGA |
| GAPDH | Forward | ACAACTTTGGTATCGTGGAAGG |
|  | Reverse | GCCATCACGCCACAGTTTC |
| G0S2 | Forward | GAAGATGGTGAAGCTGTACGT |
|  | Reverse | TGCACACAGTCTCCATCAG |
| SCARA3 | Forward | CACGAAGAGAACATGCATGATC |
|  | Reverse | CTTCCAGAGACTCAAACCTCTC |
| FXYD3 | Forward | GACGCCAATGACCTAGAAGATA |
|  | Reverse | TTTTGCACTCATGACGATGATG |
| SUSD2 | Forward | TCTTTCACTTTCACCCCAAAAC |
|  | Reverse | AATTTTTGCTGTCGATGATCCG |
| Gene Names |  | Primers sequences (5’-3’) |
| CRABP1 | Forward | GAGAACAAGATTCACTGCACAC |
|  | Reverse | ATGTCAGGATTAGCTCATCGTT |
| GRIN2D | Forward | CTTCCTGTCCTACATTGAGGTG |
|  | Reverse | ATGAACCAGACGTAGCCAGATC |
| ANKRD22 | Forward | TGCCAAGCAGCCTATCAGAATGAC |
|  | Reverse | CCTCGCCTGCAAGCACAGATC |
| ZNF264 | Forward | CCTCATTCGCCACTTCAGCATCC |
|  | Reverse | GAATCCGCTTGTGCCTCGTGAG |
| FGB | Forward | GTATTTGCTGAAAGACCTGTGG |
|  | Reverse | AATTGGTGCTTTTCCAGTTCTG |
| SLC25A35 | Forward | GGCACATACCAGCGGCACTAC |
|  | Reverse | AGTCGGATGCCATTCATCAGGAAC |
| PYROXD2 | Forward | AGATTTGGAGAGAATCTTCGGG |
|  | Reverse | CATGCTCTTGAGGTCCCTAAAG |
| DOC2A | Forward | CTCAAGCCCATGGATTTCAATG |
|  | Reverse | CCTCTGAGTCTTCGTTTTTAGC |
| PROCA1 | Forward | GCACCTACACTCTGTCAACCACTG |
|  | Reverse | TCGCTCCACATGCTCCTCCTC |
| BAHCC1 | Forward | AAGACTTTGAGTTCGACGACAA |
|  | Reverse | ATGACGATGCTATAGATGTCGG |
| HDAC5 | Forward | CTACGACACGTTCATGCTAAAG |
|  | Reverse | CACTGTCTGGATCTCATCTAGC |
| c-Myc | Forward | GTCAAGAGGCGAACACACAAC |
|  | Reverse | TTGGACGGACAGGATGTATGC |
| BCL2(for RIP) | Forward | GGCTTCTAGCGCTCGGCA |
|  | Reverse | AAGGAGCCGGGGACGGA |
