# Supplementary material for: Downregulation of Linc00173 increases BCL2 mRNA stability via the miR-1275/PROCA1/ZFP36L2 axis and induces acquired cisplatin resistance of lung adenocarcinoma
Source: J Exp Clin Cancer Res. 2023 Jan 10;42:12. doi: 10.1186/s13046-022-02560-6 (PMC9830831; doi:10.1186/s13046-022-02560-6)
Supplement: Supplementary file 9 — Additional file 9. [file 13046_2022_2560_MOESM9_ESM.docx]

**Supplementary Table 4. Correlation between LINC00173 expression and chemotherapeutic response in LUAD patients (n=129).**

| **Chemotherapy response** | **cases** | **LINC00173**  **expression** | | χ^2^ | ***p* Value** |
| --- | --- | --- | --- | --- | --- |
|  |  | Low | High |  |  |
| Platinum-resistant | 86 | 46（53.5%） | 40（46.5%） | 5.048 | **0.025** |
| Platinum-sensitive | 43 | 14（32.6%） | 29（67.4%） |  |  |

Chi-square test was applied to assess the expression of LINC00173 in platinum-resistant group and platinum- sensitive group.
